# Comparative Profiling of Free Amino Acids, Free Fatty Acids, and Volatile Organic Compounds in the *Longissimus Thoracis* Muscle of Three Sheep Breed Groups

**DOI:** 10.3390/foods15213945

**Published:** 2026-11-03

**Authors:** Mingxuan Guan, Peiling Wei, Rongyin Zhang, Liwei Zhong, Xuanyue Li, Bo Hu, Ting Chai, Ping Gong

**Affiliations:** Xinjiang Uygur Autonomous Region Academy of Animal Science, Urumqi 830011, China

**Keywords:** sheep meat, breed effect, free amino acids, free fatty acids, volatile organic compounds

## Abstract

This study investigated breed-related differences in the flavor chemistry of the longissimus thoracis muscle from Cele Black sheep (CLH), Australian White × Hu crossbred sheep (AH), and Hotan sheep (BDT). Eighteen yearling rams (six animals per group) were evaluated. Free amino acids (FAAs) were quantified by LC–MS/MS, and free fatty acids (FFAs) and volatile organic compounds (VOCs) were analyzed by GC–MS, followed by multivariate analysis, relative odor activity value (rOAV) analysis, and correlation analysis. Muscle samples from the three breeds showed comparable pH, lightness, and shear force values, whereas BDT exhibited higher redness and yellowness values. Breed-related differences were mainly observed in specific flavor-related compounds. Glutamic acid was higher in CLH (27.77 μg/g) and BDT (27.11 μg/g) than in AH (21.21 μg/g; *p* < 0.05). Stearic acid (C18:0) was higher in BDT (63.72 μg/g) than in CLH (41.10 μg/g) and AH (36.37 μg/g; *p* < 0.01). Docosatetraenoic acid (C22:4n-6) was higher in AH (9.33 μg/g) than in CLH (7.31 μg/g) and BDT (7.29 μg/g; *p* < 0.01). A total of 201 VOCs were annotated in raw-muscle headspace. Although overall VOC profiles were broadly similar among breeds, differences were observed in specific VOCs and annotated odor attributes, and rOAV analysis identified multiple compounds as potential aroma contributors. Correlation analysis followed by false discovery rate (FDR) correction showed that only Ile and Lys were significantly negatively correlated with o-xylene, whereas the associations of Tyr, C20:3n-6, and C22:4n-6 with VOCs were exploratory and did not remain significant. These co-variation patterns may reflect shared upstream metabolic or oxidative pathways rather than direct precursor–product conversion. These findings characterize breed-related differences in the flavor-associated chemical profiles of raw lamb muscle; however, their implications for cooked-meat aroma and perceived flavor require further validation through descriptive sensory analysis and cooked-meat VOC measurements.

## 1. Introduction

Lamb meat is widely valued by consumers for its high nutritional value and distinctive sensory properties. Tenderness, color, taste, and aroma are important attributes that determine lamb quality and consumer acceptance. Compared with conventional physicochemical quality traits, flavor has a more complex chemical basis. Its formation depends not only on the composition of volatile organic compounds (VOCs) but also on the combined effects of free amino acids (FAAs), fatty acids, reducing sugars, and other low-molecular-weight precursors in meat [1,2]. During heating, reactions such as the Maillard reaction, Strecker degradation, and lipid oxidation generate a wide range of volatile compounds, including aldehydes, ketones, alcohols, heterocyclic compounds, and sulfur-containing compounds. Collectively, these compounds contribute to the complex aroma characteristics of meat [3,4].

Breed is an important intrinsic factor that influences lamb quality and flavor chemistry [5]. Differences in genetic background can affect muscle development and metabolic processes, thereby affecting lipid metabolism, fatty acid composition, and the accumulation of flavor-related metabolites. These changes may contribute to variations in meat quality and flavor precursor profiles [6,7]. Comparative studies among sheep breeds have shown that breed can significantly affect fatty acid, amino acid, and VOC profiles [8]. Previous research has also demonstrated that sheep breeds and muscle types can be differentiated on the basis of their flavor precursor and VOC profiles. Moreover, certain amino acids and fatty acids are closely associated with the formation of volatile compounds [9]. Crossbreeding Australian White sheep with Hu sheep has also been shown to alter meat quality as well as amino acid, fatty acid, and volatile flavor compound profiles. These findings suggest that crossbred and indigenous sheep may exhibit distinct flavor-related chemical phenotypes [10]. However, systematic comparisons of flavor-related chemical profiles among indigenous sheep breeds and crossbreds remain relatively limited, especially when FAAs, free fatty acids (FFAs), and headspace VOCs are integrated. The three breed groups compared here were selected because of their distinct genetic and production backgrounds. Cele Black sheep (CLH) and Hotan sheep (BDT) are indigenous sheep genetic resources in China that are adapted to local environments and contribute to local lamb production and genetic diversity. In contrast, Australian White × Hu crossbred sheep (AH) represent a crossbred group that combines the growth and carcass characteristics of Australian White sheep with the reproductive and adaptive traits of Hu sheep, and is therefore of commercial interest for lamb production. Comparing these groups under a common feeding and management regimen provides a comparable basis for evaluating breed-related differences in flavor-related chemical profiles. To our knowledge, integrated comparisons of FAAs, FFAs, headspace VOCs, and relative odor activity values (rOAVs) among CLH, AH, and BDT remain limited. The present study therefore aimed to characterize their flavor-related chemical differences and to provide information relevant to the quality-oriented utilization of indigenous and crossbred sheep resources.

FAAs and FFAs are important non-volatile flavor precursors in meat, and variations in their composition are closely associated with the resulting volatile flavor profiles [11]. Glutamic acid (Glu) and aspartic acid (Asp) can directly contribute to umami taste, whereas alanine (Ala) and glycine (Gly) are associated with sweetness. Amino acids can also participate in the Maillard reaction and Strecker degradation to generate a variety of meat aroma compounds. In addition, the oxidation of unsaturated fatty acids produces VOCs such as aldehydes, alcohols, ketones, and furans, which are important contributors to fatty notes and characteristic meat aromas [1]. Therefore, comparisons based solely on the identities or relative abundances of VOCs may not fully account for breed-related differences in flavor. Furthermore, highly abundant VOCs do not necessarily make substantial contributions to aroma, because their sensory impact also depends on their odor thresholds. The rOAV, which relates the concentration of a compound to its odor threshold, is an important tool for screening potentially important aroma-active compounds [12]. Previous research has shown that the overall aroma of lamb is generally shaped by the combined contributions of multiple compounds with high OAVs rather than by a single highly abundant volatile compound [13].

Accordingly, this study investigated the *longissimus thoracis* muscles from CLH, AH, and BDT. Meat quality and the profiles of FAAs, FFAs, and VOCs were compared among the three groups. rOAV analysis and multivariate analysis were combined to screen for potentially important aroma-active compounds. Potential associations between flavor precursors and key VOCs were further explored. This study aimed to characterize breed-related differences in lamb flavor chemistry and identify potential flavor-related compounds associated with these differences. The findings may provide a scientific basis for meat quality evaluation, characterization of distinctive flavor attributes, and quality-oriented utilization of indigenous and crossbred sheep resources.

## 2. Materials and Methods

### 2.1. Experimental Animals and Sample Collection

All animal experiments and slaughter procedures were approved by the Science and Technology Ethics Committee of Xinjiang Academy of Animal Science and conducted in accordance with its animal ethics and welfare regulations (Approval No. 202510012). A total of 18 healthy rams (12.0 ± 0.5 months of age) were used, comprising six CLH (38.55 ± 1.73 kg), six AH (39.08 ± 2.11 kg), and six BDT (38.19 ± 1.92 kg) sheep. Before the 52-day trial, all animals were reared under the same conventional indoor feeding and management system at the same farm, with ad libitum access to water, and no breed-specific differences in feeding or management were applied. During the 52-day trial, including a 7-day adaptation period and a 45-day experimental period, all animals were raised under identical environmental and management conditions at Xinjiang Jinken Aokun Agricultural and Animal Husbandry Technology Co., Ltd. (Hotan, China). Throughout the trial, all sheep received the same pelleted diet with ad libitum access to feed and water. Feed offered and refusals were recorded daily during the 45-day experimental period, and average daily feed intake (ADFI) was calculated on a dry matter basis. The ADFI was comparable among the three groups (CLH: 1.60 ± 0.12 kg/d; AH: 1.77 ± 0.36 kg/d; BDT: 1.81 ± 0.55 kg/d). The dietary composition and nutrient levels are provided in Supplementary Table S1.

At the end of the feeding trial, all animals were fasted for 12 h and deprived of water for 2 h before slaughter. Animals were stunned by electrical stunning and immediately slaughtered by exsanguination via severing the carotid arteries and jugular veins. Slaughter was performed in a randomized order across the three breed groups to minimize batch effects. After exsanguination, carcasses were immediately transferred to a chilling room at 4 °C. Within 30 min postmortem and before complete chilling, longissimus thoracis samples were collected between the 11th and 12th thoracic vertebrae. After removal of visible surface fat, fascia, and connective tissue, the samples were divided into portions, immediately frozen in liquid nitrogen, and stored at −80 °C for subsequent free amino acid, free fatty acid, and VOC analyses.

### 2.2. Meat Quality Measurements

At 45 min postmortem, the pH and surface color of the *longissimus thoracis* muscle were measured using a portable pH meter with a penetrating electrode (PH818M, SMART SENSOR, Dongguan, China) and a portable colorimeter (NR10QC, 3nh, Shenzhen, China), respectively. Due to practical constraints during the experiment, only pH at 45 min postmortem was measured, and ultimate pH at 24 h postmortem was not determined. The pH meter was calibrated before each measurement session using standard buffer solutions of pH 4.00, 6.86, and 9.18 at room temperature, with automatic temperature compensation. The electrode was rinsed with deionized water and blotted dry between measurements. The colorimeter was calibrated using the supplied white reference plates according to the manufacturer’s instructions before each measurement session. Color measurements were performed on freshly cut muscle surfaces that were exposed to ambient air, and excess surface moisture was gently blotted with filter paper before measurement. The instrument was set to the CIE L*a*b color space with a D65 illuminant, an 8°/d measurement geometry, a Φ4-mm measuring aperture, and a 10° standard observer. For each sample, three readings were taken at different locations on the muscle surface, and the average L, a, and b* values were reported. Fresh longissimus thoracis muscle samples (approximately 6 cm × 4 cm × 3 cm) were heated in an 80 °C water bath until the internal temperature reached 70 °C and held at 70 °C for 0 min (i.e., removed immediately upon reaching 70 °C). After cooking, the samples were cooled at room temperature for approximately 30 min and then refrigerated at 4 °C overnight (approximately 14 h) before measurement. Cylindrical cores (diameter: 1.3 cm, length: approximately 1.5 cm) were then removed parallel to the muscle fiber direction to obtain standardized samples. A minimum of 6 cores were evaluated per animal, and the average value was used for statistical analysis. Shear force was measured using a C-LM3B digital meat tenderness meter (Tenovo, Beijing, China) at a cutting speed of 5 mm/s. The water loss rate was determined using a modified filter-paper press-loss method [14]. Briefly, a 1-cm-thick muscle slice was excised perpendicular to the muscle fiber direction, and a circular sample (3 cm in diameter) was obtained using a DL-100 sampler (Sumspring, Jinan, China). The sample was placed between six layers of filter paper on each side and subjected to a load of 35 kg for 5 min. The sample was weighed before (W1) and after (W2) compression. The water loss rate was calculated as follows: Water loss rate (%) = [(W1 − W2)/W1] × 100. The same compression conditions were applied to all samples to ensure consistency.

### 2.3. Determination of Free Amino Acids

#### 2.3.1. Sample Preparation

Muscle samples were removed from storage at −80 °C and placed on dry ice. Approximately 50 mg of each sample was weighed into a 2 mL centrifuge tube containing a steel bead. Subsequently, 250 μL of precooled 70% aqueous methanol containing 5 mmol/L iodoacetamide (IAM) and [^2^H_2_]-L-threonine as the internal standard (1 μg/mL) was added. The mixture was vortexed for 5 min in the dark, sonicated for 10 min, and allowed to stand at room temperature for 30 min. Another 250 μL of precooled 70% aqueous methanol was then added. The mixture was vortexed for 5 min and centrifuged at 12,000 rpm for 5 min at 4 °C. A 300 μL aliquot of the supernatant was transferred and incubated at −20 °C for 30 min, followed by centrifugation at 12,000 rpm for 10 min at 4 °C. Finally, 200 μL of the supernatant was passed through a protein precipitation plate, and the filtrate was used for LC–MS/MS analysis.

#### 2.3.2. LC–MS/MS Conditions

FAAs were analyzed using an ExionLC™ AD UHPLC system coupled to a QTRAP^®^ 6500+ mass spectrometer (SCIEX, Framingham, MA, USA) with an ACQUITY BEH Amide column (100 mm × 2.1 mm, 1.7 μm; Waters, Milford, MA, USA). Mobile phases A and B were water and acetonitrile, respectively, each containing 2 mmol/L ammonium acetate and 0.04% formic acid. The gradient was programmed as follows: 0–1.2 min, 90% B; 1.2–9.0 min, 90–60% B; 9.0–10.0 min, 60–40% B; 10.0–11.0 min, 40% B; and 11.01–15.0 min, 90% B for re-equilibration. The flow rate, column temperature, and injection volume were 0.4 mL/min, 40 °C, and 2 μL, respectively. Mass spectrometric detection was performed using electrospray ionization (ESI) at 550 °C, with spray voltages of 5500 and −4500 V in positive and negative ion modes, respectively, and a curtain gas pressure of 35 psi. Data were acquired in multiple reaction monitoring (MRM) mode, with the declustering potential (DP) and collision energy (CE) optimized for each target compound. Compound-specific MRM transitions, retention times, and identification evidence are provided in Supplementary Table S3.

#### 2.3.3. Quantification and Analytical Validation

Targeted amino acids and related metabolites were identified based on retention times and MRM transitions of authentic standards (purity > 99%) and quantified using calibration curves. Calibration standards were prepared at 1, 2, 5, 10, 20, 50, 100, 200, 500, 1000, 2000, 5000, 10,000, and 20,000 ng/mL. Calibration curves were constructed by plotting the peak area ratio of each analyte to the internal standard against the concentration ratio of the external standard to the internal standard. The internal standard was added at 1 μg/mL during sample preparation. The lower and upper limits of quantification (LLOQ and ULOQ) corresponded to the lowest and highest calibration concentrations (1 and 20,000 ng/mL, respectively), within which accurate quantification was achieved. Sample concentrations were calculated as follows: content (ng/g) = c × V/1000/m, where c is the concentration obtained from the calibration curve (ng/mL), V is the extraction volume (μL), and m is the sample weight (g). Quality control (QC) samples were prepared from a pooled mixture and injected every 10 samples. The QC CV distribution showed that more than 80% of metabolites had CV < 0.3, indicating acceptable analytical stability. Please refer to Table S3 for specific information.

### 2.4. Determination of Free Fatty Acids

#### 2.4.1. Sample Preparation

Muscle samples were ground in liquid nitrogen using a ball mill at 30 Hz for 1.5 min. Approximately 50 mg of each sample was extracted with 700 μL of methanol containing isotope-labeled internal standards (2 μg/mL C8:0-d15, 2 μg/mL C11:0-d21, 2 μg/mL C17:0-d33, 5 μg/mL 13C18:2n6c, and 5 μg/mL C22:0-d43) by vortexing for 10 min and sonication at 4 °C for 15 min, followed by centrifugation at 4200 rpm for 5 min at 4 °C. A 500 μL aliquot of the supernatant was mixed with 200 μL of 0.05 mol/L NaOH in methanol and evaporated to dryness under nitrogen. The residue was mixed with 400 μL of 0.4 mol/L NaOH in methanol and incubated at 70 °C for 10 min. After cooling, 500 μL of dichloromethane and 200 μL of water were added, followed by mixing and centrifugation. The organic phase was collected and mixed with 300 μL of 2 mol/L HCl in methanol for methylation at 70 °C for 20 min. After cooling, the mixture was extracted with 500 μL of n-hexane and 300 μL of water. Following centrifugation, 200 μL of the n-hexane layer was collected for GC–MS analysis.

#### 2.4.2. GC–MS Conditions

FFAs were analyzed using a GC–MS system equipped with a DB-5MS capillary column (30 m × 0.25 mm i.d., 0.25 μm film thickness; Agilent J&W Scientific, Folsom, CA, USA). Helium (≥99.999%) was used as the carrier gas at 1.0 mL/min, with a split ratio of 5:1 and an injection volume of 2 μL. The injector and transfer line temperatures were 250 and 280 °C, respectively. The oven temperature was programmed from 50 °C (1.5 min) to 170 °C at 30 °C/min, 200 °C at 15 °C/min, 220 °C at 5 °C/min (2 min), 228 °C at 8 °C/min, 240 °C at 20 °C/min (2 min), 265 °C at 25 °C/min (2 min), and finally 280 °C at 30 °C/min (2 min). Mass spectrometric detection was performed using electron ionization at 70 eV, with ion source and quadrupole temperatures of 230 and 150 °C, respectively. Data were acquired in selected ion monitoring (SIM) mode. For each compound, one quantitative ion and two to three qualitative ions were selected. Identification was based on matching retention times and ion abundance ratios with reference standards.

#### 2.4.3. Calibration, Quantification, and Analytical Validation

FFAs were quantified using calibration curves. Calibration standards were prepared at 0.004, 0.01, 0.02, 0.04, 0.1, 0.2, 0.4, 1, 2, 4, 10, 15, and 20 μg/mL, each containing the same isotope-labeled internal standards as the samples. Calibration curves were constructed by plotting the peak area ratio of each analyte to the internal standard against the concentration ratio of the external standard to the internal standard. The LLOQ and ULOQ corresponded to the lowest and highest calibration concentrations (0.004 and 20 μg/mL, respectively), within which accurate quantification was achieved. Sample concentrations were calculated as follows: content (μg/g) = c × v3 × v1/v2/m/1000, where c is the concentration obtained from the calibration curve (μg/mL), v1 is the extraction volume (μL), v2 is the supernatant volume collected (μL), v3 is the redissolution volume (μL), and m is the sample weight (g). QC samples were prepared from a pooled mixture and injected every 10 samples. The QC CV distribution showed that more than 80% of metabolites had CV < 0.3, indicating acceptable analytical stability.

### 2.5. Volatile Organic Compound Analysis

#### 2.5.1. Headspace Solid-Phase Microextraction

Muscle samples were ground in liquid nitrogen and homogenized. Approximately 0.20 g of homogenized raw muscle was placed in a headspace vial containing 0.20 g of NaCl and 20 μL of 3-hexanone-2,2,4,4-d_4_ internal standard solution (10 μg/mL in n-hexane). After equilibration at 60 °C for 5 min with agitation, headspace VOCs were extracted at 60 °C for 15 min using a 120 μm divinylbenzene/carbon wide range/polydimethylsiloxane (DVB/CWR/PDMS) fiber. No separate cooking treatment was applied to the muscle samples before HS-SPME analysis.

#### 2.5.2. GC–MS Analysis

VOCs were analyzed using a GC–MS system equipped with a DB-5MS capillary column (30 m × 0.25 mm i.d., 0.25 μm film thickness; Agilent J&W Scientific, USA). Helium (≥99.999%) was used as the carrier gas at 1.2 mL/min, and the injector temperature was 250 °C. The oven temperature was programmed from 40 °C (3.5 min) to 100 °C at 10 °C/min, 180 °C at 7 °C/min, and 280 °C at 25 °C/min, with a final hold of 5 min. Mass spectrometric detection was performed using electron ionization at 70 eV, with ion source, quadrupole, and MS interface temperatures of 230, 150, and 280 °C, respectively. Data were acquired in selected ion monitoring (SIM) mode. For each compound, one quantitative ion and two to three qualitative ions were selected according to GB 23200.8-2016 [15]. Identification was based on matching retention times and ion abundance ratios with those of reference standards or the in-house database.

#### 2.5.3. Semiquantitative Analysis and rOAV Calculation

Analytical precision was assessed using QC samples prepared from a pooled mixture and injected every 10 samples. The CV distribution showed that more than 85% of metabolites had CV < 0.5 and more than 75% had CV < 0.3, indicating acceptable analytical stability.

VOCs were semiquantified using the internal standard method [16], as absolute calibration curves were not established for all compounds. The relative concentration of each compound was calculated as follows:

$$C_{i}=\frac{V_{s}\times C_{s}}{M}\times \frac{I_{i}}{I_{s}}\times 10^{-3}$$

where *C_i_* is the relative concentration of compound *i* (μg/g), *V_s_* is the volume of internal standard added (20 μL), *C_s_* is the concentration of the internal standard (10 μg/mL), *M* is the sample weight (g), *I_i_* is the peak area of compound *i*, and *I_s_* is the peak area of the internal standard.

For the rOAV:

$$\mathrm{rOA}V_{i}=\frac{C_{i}}{T_{i}}$$

where *rOAV_i_* is rOAV of compound *i* (dimensionless), *C_i_* is relative concentration of compound *i* (μg/g), *T_i_* is odor threshold of compound *i* in the corresponding matrix (μg/g) [17]. Compounds with *rOAV* ≥ 1 were considered potential contributors to the overall aroma.

### 2.6. Statistical Analysis

All data are expressed as the mean ± standard error of the mean (SEM). For variables satisfying the assumptions of normality and homogeneity of variance, differences among breeds were analyzed using one-way analysis of variance (ANOVA), followed by Duncan’s multiple range test. Differences were considered statistically significant at *p* < 0.05. Associations among FAAs, FFAs, and key VOCs were evaluated using Spearman’s correlation analysis. For correlation analysis, *p* values were adjusted for multiple testing using the false discovery rate (FDR) method (Benjamini–Hochberg procedure). Statistical analysis was performed using IBM SPSS Statistics 26.0 (IBM Corp., Armonk, NY, USA). Figures were generated using GraphPad Prism 10.1.2 (GraphPad Software, La Jolla, CA, USA) and formatted using Adobe Illustrator 2025 (Adobe Inc., San Jose, CA, USA).

## 3. Results

### 3.1. Meat Quality Characteristics of Different Sheep Breeds

The meat quality characteristics of the *longissimus thoracis* muscle from different sheep breeds are presented in Figure 1. No significant differences were observed in pH or lightness (L*) among CLH, AH, and BDT (*p* > 0.05), indicating comparable values for these quality traits across the three breeds (Figure 1A,B). In contrast, redness (a*) and yellowness (b*) differed significantly among breeds (Figure 1C,D). BDT exhibited a significantly higher a* value than CLH and AH, while CLH also had a higher a* value than AH (*p* < 0.01). The b* values were significantly higher in BDT than in CLH and AH (*p* < 0.01), with no significant difference between the latter two groups. The water loss rate was significantly higher in CLH than in AH (*p* < 0.05), whereas no significant differences in shear force were observed among the three breeds (*p* > 0.05; Figure 1E,F).

### 3.2. Free Amino Acid Profiles and Breed-Related Differences

FAAs and related metabolites in the *longissimus thoracis* muscle from the three breeds were quantified using LC–MS/MS. More than 80% of metabolites in the QC samples exhibited coefficients of variation (CVs) below 0.20 (Figure 2A), indicating good analytical repeatability. Principal component analysis (PCA) showed no clear separation among the three breed groups, whereas the supervised OPLS-DA score plot suggested greater separation, particularly between AH and CLH (Figure 2B,C). The model yielded R^2^X = 0.276, R^2^Y = 0.890, and Q^2^ = 0.592. The 200-permutation test yielded *p* < 0.05 (Supplementary Figure S1A), providing support for the observed supervised pattern. Nevertheless, the OPLS-DA result was interpreted as exploratory rather than as evidence of definitive breed classification. Pairwise comparisons identified a limited number of differential metabolites. Specifically, 1, 5, and 3 metabolites were increased, whereas 2, 5, and 6 metabolites were decreased in the AH vs. BDT, AH vs. CLH, and BDT vs. CLH comparisons, respectively (Supplementary Figure S1B–D). These results suggest that breed-related differences were mainly associated with specific metabolites rather than extensive changes in the overall amino acid profiles. Detailed information on the differential metabolites is provided in Supplementary Table S2. In the variable importance in projection (VIP) analysis of the three breeds, 2-aminoethanesulfonic acid (taurine) showed the highest contribution to breed discrimination (VIP = 2.30), followed by N,N-dimethylglycine (VIP = 1.98), trimethylamine N-oxide (VIP = 1.93), and Glu (VIP = 1.88) (Figure 2D). Taurine showed a higher relative abundance in AH, whereas trimethylamine N-oxide was more abundant in CLH (Figure 2E). These metabolites were therefore identified as major contributors to the discrimination of amino acid metabolic profiles among breeds.

Among the 20 FAAs analyzed, Glu, isoleucine (Ile), glutamine (Gln), cysteine (Cys), Ala, lysine (Lys), and tyrosine (Tyr) differed significantly among breeds (Table 1). Glu content was significantly higher in CLH and BDT than in AH (*p* < 0.05), whereas Asp showed no significant difference. Accordingly, the total umami amino acid (UAA) contents were significantly higher in CLH and BDT than in AH (*p* < 0.05). For sweet amino acids, CLH exhibited a significantly higher Ala content than BDT and had the highest total sweet amino acid (SAA) content (*p* < 0.05). In addition, Gln content was significantly higher in CLH than in AH. BDT exhibited a higher Ile content than AH and higher Lys and Cys contents than CLH. Tyr content was significantly higher in AH and BDT than in CLH (*p* < 0.05). No significant differences were observed in the total essential amino acid (EAA) content among the three breeds (*p* > 0.05). Overall, breed-related differences in FAAs were mainly associated with specific taste-active amino acids and related metabolites rather than total EAA content. CLH exhibited higher SAA and UAA contents, whereas BDT showed higher levels of certain branched-chain and sulfur-containing amino acids. These differences suggest that breed-related variations in free amino acid composition may contribute to differences in taste-related chemical profiles among breeds.

### 3.3. Free Fatty Acid Profiles and Breed-Related Differences

GC–MS analysis showed that more than 80% of the detected FFAs exhibited CVs below 0.20 in the QC samples, indicating good analytical repeatability (Figure 3A). PCA showed no clear separation among the three breed groups, whereas the supervised OPLS-DA score plot suggested partial group separation (Figure 3B,C). The model yielded R^2^X = 0.546, R^2^Y = 0.828, and Q^2^ = 0.393. In the 200-permutation test, the permutation *p* values for Q^2^ and R^2^Y were 0.020 and 0.165, respectively (Figure 3D). These results suggest that breed-related differences existed in the free fatty acid profiles, although the overall separation was limited.

One-way ANOVA revealed that significant breed-related differences were mainly observed in C6:0, C18:0, C19:0, C20:3n-6, and C22:4n-6 (Table 2). The C6:0 content was significantly higher in CLH than in AH and BDT, whereas the C18:0 content was significantly higher in BDT than in CLH and AH (*p* < 0.05). C19:0 also differed significantly among breeds (*p* = 0.008), with AH showing a lower content than CLH and BDT. Among polyunsaturated fatty acids (PUFAs), AH exhibited a significantly higher C20:3n-6 content than CLH, while its C22:4n-6 content was significantly higher than that in both CLH and BDT (*p* < 0.05). In contrast, no significant differences were observed in C18:2n-6, C18:3n-3, C20:4n-6, C22:5n-3, or C22:6n-3 among the three breeds. The total concentrations of saturated fatty acids (SFAs), monounsaturated fatty acids (MUFAs), and PUFAs did not differ significantly among breeds (*p* > 0.05). Although BDT exhibited a numerically higher SFA content, the difference was not statistically significant (*p* = 0.075). These findings indicate that breed-related differences in free fatty acid composition were mainly associated with changes in specific fatty acids rather than overall SFA, MUFA, or PUFA contents. In particular, BDT exhibited a higher C18:0 content, whereas AH showed higher levels of C20:3n-6 and C22:4n-6.

### 3.4. Volatile Organic Compound Profiles and Breed-Related Differences

A total of 201 VOCs were annotated using GC–MS. Detailed identification information, relative abundances in each breed group, and statistical results for all 201 VOCs are provided in Supplementary Table S4. These included 65 aldehydes, ketones, and esters; 62 hydrocarbons; 28 alcohols and amines; 11 heterocyclic compounds; 13 terpenes; and smaller numbers of benzene and its derivatives, organic acids and their derivatives, and ethers (Figure 4A). Hydrocarbons were the most abundant VOC class in all three breeds, accounting for 58.75%, 65.23%, and 63.69% of the total VOC relative abundance in CLH, AH, and BDT, respectively. The combined relative abundance of aldehydes, ketones, and esters was highest in CLH (21.59%) and the lowest in AH (13.39%). In contrast, AH exhibited a high relative abundance of alcohols and amines (15.23%) (Figure 4B). These results indicate that the three breeds had similar major VOC classes but differed in their relative compositions.

More than 75% of VOCs in the QC samples exhibited CVs below 0.30, indicating good analytical repeatability (Supplementary Figure S2A). In the PCA model, PC1 and PC2 explained 43.71% and 17.63% of the total variance, respectively, and no clear separation was observed among the three breeds (Figure 4C). To apply a consistent supervised strategy, pairwise OPLS-DA was subsequently used for the predefined breed comparisons (Supplementary Figure S2B). The pairwise OPLS-DA models showed different validation performances. For AH vs. BDT, the model yielded R^2^X = 0.705, R^2^Y = 0.515, and Q^2^ = 0.130, and the 200-permutation test was not significant (*p* > 0.05), indicating that the apparent supervised separation was not supported by permutation validation (Supplementary Figure S2C). In contrast, the AH vs. CLH model yielded R^2^X = 0.683, R^2^Y = 0.830, and Q^2^ = 0.511, with a significant permutation test (*p* < 0.05), providing comparatively stronger support for this supervised separation (Supplementary Figure S2D). For BDT vs. CLH, the model yielded R^2^X = 0.861, R^2^Y = 0.992, and Q^2^ = 0.455; however, the permutation test was not significant (*p* > 0.05), and the apparent separation was therefore not considered adequately validated (Supplementary Figure S2E). Overall, the supervised OPLS-DA models were used as exploratory tools to visualize potential breed-related variation rather than as evidence of robust breed classification.

VIP analysis further revealed that a limited number of VOCs contributed substantially to breed discrimination. In the overall comparison, 2,6,10-dodecatrien-1-ol, 3,7,11-trimethyl-, acetate, (E,E)- had the highest VIP score. Other compounds with relatively high contributions included undecane, 4,8-dimethyl-; decane, 6-ethyl-2-methyl-; and 2,7-dimethyl-1-octanol (Figure 4D). In the AH vs. CLH comparison, cyclohexanol, 4-methyl-, trans- had the highest VIP score, followed by 2,6,10-dodecatrien-1-ol, 3,7,11-trimethyl-, acetate, (E,E)- and 4-heptyn-2-ol (Figure 4D). Hierarchical clustering analysis showed that several VOCs with high VIP scores exhibited higher standardized relative abundances in AH than in CLH. This pattern was consistent with the separation between AH and CLH observed in the supervised models (Figure 4E).

### 3.5. Potential Aroma-Active Compounds and Their Odor Attributes

To evaluate the potential contributions of individual VOCs to the overall aroma, rOAVs were calculated based on compound abundance and odor thresholds. The overall rOAV distributions were relatively similar among the three breeds, and more than 10 compounds with rOAV ≥ 1 were detected in each group (Figure 5A). These results suggest that the three breeds shared several potentially important aroma-active compounds, although the relative contributions of individual compounds varied. Odor attribute annotation of differential VOCs further revealed potential aroma differences among breeds (Figure 5B). In the overall comparison, the woody attribute was the most frequently annotated (n = 5), followed by green and herbal (n = 4). Differential VOCs between AH and BDT were mainly associated with green (n = 11), fruity (n = 9), and fatty (n = 7) attributes. In the AH vs. CLH comparison, green (n = 7) and waxy (n = 5) were the most frequent attributes, followed by cucumber and fatty (n = 4). In contrast, fewer differential VOCs with annotated odor attributes were observed between BDT and CLH, suggesting relatively limited differences in their annotated volatile flavor profiles. The Sankey diagram further illustrated the relationships between differential VOCs and their associated odor attributes (Figure 5C). In both the AH vs. BDT and AH vs. CLH comparisons, most differential VOCs showed decreased relative abundances in AH. In contrast, the BDT vs. CLH comparison involved fewer differential compounds and associated odor attributes.

Overall, the multivariate analysis, rOAV evaluation, and odor attribute annotation indicated that the three breeds shared similar major VOC classes. However, breed-related differences in potential odor characteristics were mainly associated with variations in the relative abundances of specific VOCs with potentially high aroma contributions rather than changes in the overall number of VOC classes. It should be noted that odor attributes were assigned based on database and literature descriptions, and VOCs were measured in raw frozen muscle. Therefore, these results should be interpreted as potential odor characteristics under the specified analytical conditions rather than cooked-meat aroma or experimentally verified sensory perceptions. Their sensory relevance requires further validation through descriptive sensory analysis and cooked-meat VOC measurements.

### 3.6. Associations Between Non-Volatile Flavor-Related Compounds and Potential Aroma-Active VOCs

Spearman correlation analysis was performed to investigate potential associations among FAAs, FFAs, and key VOCs (Figure 6). To control for multiple testing, *p* values were adjusted using the false discovery rate (FDR) method, and only associations that remained significant after FDR correction were reported. After FDR correction, only Ile and Lys were significantly negatively correlated with o-xylene (*p* < 0.05). The other correlations observed in the unadjusted analysis, including those between Tyr, C20:3n-6, C22:4n-6 and various VOCs, did not remain statistically significant after FDR correction and should therefore be regarded as exploratory findings. These results indicate that, among the non-volatile flavor-related compounds analyzed, Ile and Lys showed the most robust statistical co-variation with o-xylene, although the underlying mechanisms require further investigation.

## 4. Discussion

### 4.1. Breed-Related Differences in Meat Quality Characteristics

Meat color and pH are important indicators of postmortem meat quality. The pH reflects postmortem glycolysis and is associated with changes in muscle protein structure, whereas meat color directly influences consumers’ perceptions of freshness and quality [18,19]. In the present study, the three sheep breeds exhibited generally similar meat quality characteristics, but significant differences were observed in meat color. No significant differences were found in pH, L* value, or shear force among the three breeds, whereas BDT exhibited higher a* and b* values. These findings indicate that breed-related differences in the measured meat quality traits were mainly observed in color, while differences in postmortem acidification and instrumental tenderness were relatively limited. Meat color is primarily determined by myoglobin content and its chemical state. It is also regulated by several postmortem biochemical processes, including mitochondrial oxygen consumption, metmyoglobin-reducing activity, and oxidative reactions [19,20]. Previous studies have shown that breed and production conditions can affect lamb color and color stability independently of intrinsic muscle characteristics such as pH and myoglobin concentration [21,22]. A recent study found that significant changes in lamb meat color occurred without a corresponding difference in pH, further indicating that variation in meat color may occur independently of postmortem acidification [23]. Therefore, because pH did not differ significantly among the three breeds in the present study, the higher a* value observed in BDT is unlikely to be explained solely by differences in postmortem acidification and may instead be associated with breed-related variation in myoglobin characteristics, muscle redox status, or other metabolic factors.

### 4.2. Free Amino Acids and Fatty Acids as Flavor-Related Compounds and Precursors

FAAs contribute directly to the basic taste of meat and serve as important precursors for volatile flavor formation during thermal processing [24,25]. In the present study, CLH and BDT exhibited higher Glu and total UAA contents than AH. CLH also had the highest total SAA content, mainly because of its higher Ala content. Glu is a typical umami amino acid, whereas Gly and Ala are generally associated with sweetness. These amino acids have also been identified as important taste-related compounds in lamb [26]. Therefore, the higher UAA and SAA contents in CLH may provide a stronger chemical basis for umami and sweet taste characteristics. Similarly, the higher Glu content in BDT may contribute to its umami-related chemical characteristics. However, free amino acid concentrations do not fully reflect perceived taste intensity. Their actual contributions also depend on taste thresholds and synergistic interactions with other taste-active compounds, such as inosine 5′-monophosphate (IMP) [26,27]. In addition to their direct contributions to taste, FAAs participate in the Maillard reaction and Strecker degradation to generate aldehydes and nitrogen- and sulfur-containing volatile compounds. Cys and Met are particularly important precursors to sulfur-containing compounds associated with meat aroma [4]. In this study, BDT exhibited relatively high Cys and Glu contents, whereas CLH showed higher Gln and Ala levels. These findings indicate that the three breeds differed in their amino acid precursor profiles, which may influence the formation of volatile compounds during subsequent thermal reactions [9]. The absence of significant differences in total EAA contents further suggests that breed-related effects were mainly associated with specific FAAs rather than the overall EAA pool. In addition, taurine, N,N-dimethylglycine, and trimethylamine N-oxide exhibited high VIP scores, indicating substantial contributions to the discrimination of metabolic profiles among breeds. However, these compounds should be considered candidate differential metabolites rather than key taste-active compounds because their sensory contributions were not verified through taste threshold measurements or sensory evaluation.

FFAs represent another important class of aroma precursors [28]. In the present study, the total contents of SFAs, MUFAs, and PUFAs did not differ significantly among the three breeds. Instead, significant differences were mainly observed in individual fatty acids, including C18:0, C20:3n-6, and C22:4n-6. These findings suggest that breed-related effects were more evident in variations in specific FFAs than in changes in the overall fatty acid classes [29,30]. During heating and oxidation, unsaturated fatty acids can generate volatile compounds, including aldehydes, alcohols, ketones, and furans. These compounds may further interact with Maillard reaction intermediates and influence the overall aroma characteristics of meat [31]. Previous studies have shown that differences in amino acid and fatty acid precursors among sheep breeds can be accompanied by changes in volatile compound profiles [32,33]. Therefore, the higher C20:3n-6 and C22:4n-6 contents in AH may represent a distinct lipid precursor profile associated with its VOC profile. However, the specific precursor–product relationships require further verification using controlled model systems and stable-isotope tracing.

The three breed groups also differed in genetic background: CLH and BDT are local breeds that may possess unique metabolic characteristics, whereas AH is a crossbred group that may exhibit heterosis. This distinction is important because the phenotype of a crossbred population may reflect not only the additive genetic contributions of the parental breeds but also heterosis and complementary effects between parental genetic backgrounds. Studies of crossbred sheep populations have revealed differences in muscle growth, lipid metabolism, and metabolite profiles, with transcriptomic and metabolomic alterations involving fatty acid and lipid metabolic pathways, PPAR signaling, AMPK signaling, and glycerophospholipid metabolism [34]. However, as no genetic, transcriptomic, or enzymatic analyses were performed in this study, these interpretations remain speculative and warrant further investigation using transcriptomic, genomic, or metabolic flux analyses.

### 4.3. Breed-Specific VOC Profiles and Key Aroma-Active Compounds

A total of 201 VOCs were annotated in the present study. Hydrocarbons were the most abundant chemical class in all three breeds, indicating broadly similar overall patterns of VOC composition. Nevertheless, the relative proportions of individual chemical classes differed among breeds. Notably, the relative abundance of VOCs does not directly reflect their contributions to aroma [35]. Most aliphatic hydrocarbons have relatively high odor thresholds and may therefore contribute little to the overall aroma of lamb, even when present at high relative abundances [36]. In contrast, certain short-chain aliphatic aldehydes and nitrogen- or sulfur-containing heterocyclic compounds generally have low odor thresholds and are important aroma-active compounds in cooked meat [37]. Thus, aroma differences among sheep breeds may depend more on the composition and concentrations of specific low-threshold VOCs than on the overall abundance of a particular chemical class [5,38]. To further evaluate the potential aroma contributions of individual VOCs, rOAVs were used for screening. Each breed contained more than 10 VOCs with rOAV values of 1 or greater, indicating that multiple compounds may potentially contribute to the overall aroma. This finding suggests that the three breeds may share a group of potential aroma-active compounds, although the relative contributions of individual compounds differed. Previous research has demonstrated that the overall aroma of lamb is generally determined by the combined effects of multiple compounds with high OAVs rather than by a single highly abundant volatile compound [13]. In the present study, PCA showed no clear natural separation among the VOC profiles of the three breeds, whereas OPLS-DA revealed a relatively distinct separation between AH and CLH. These findings suggest that the overall volatile compositions of the three breeds were relatively similar, while breed-related differences may have been concentrated in specific VOCs. Similarly, differences in certain aldehydes and other VOCs have been reported among sheep breeds and have been associated with breed-related variations in flavor precursor composition [8].

Odor attribute annotation further revealed that differential VOCs between AH and the other two breeds were more frequently associated with green, waxy, fruity, fatty, and cucumber-like attributes. In contrast, fewer differences in annotated odor attributes were identified between BDT and CLH. These findings further indicate breed-related variations in volatile flavor composition. However, odor descriptions obtained from databases or the previous literature only indicate the potential sensory properties of individual compounds. They do not necessarily represent how they are actually perceived within the lamb meat matrix. Gas chromatography–olfactometry (GC–O) combines chromatographic separation with human olfactory responses and is an important technique for identifying aroma-active compounds and characterizing their sensory attributes [39]. HS-SPME was performed at 60 °C on uncooked muscle, and no extraction-temperature or time-course control experiments were conducted. Thermally induced artifacts (e.g., lipid oxidation) cannot therefore be excluded, and the detected VOCs should be interpreted as headspace compounds obtained under these analytical conditions rather than as the native volatile profile of raw muscle. Because no cooked samples or sensory evaluation were included, the annotated odor attributes do not directly establish the perceived aroma of cooked lamb; future studies should compare raw and cooked samples and integrate targeted quantification, GC–O, and sensory evaluation.

### 4.4. Associations Between Non-Volatile Precursors and VOCs

Spearman correlation analysis was performed to investigate potential associations among FAAs, FFAs, and key VOCs. To control for multiple testing, *p* values were adjusted using the false discovery rate (FDR) method, and only associations that remained significant after FDR correction were reported. After FDR correction, only Ile and Lys were significantly negatively correlated with o-xylene. These co-variation patterns may reflect shared upstream metabolic or oxidative pathways, or breed-related differences in non-volatile compound composition, rather than evidence of direct precursor–product conversion. Previous studies have also reported close associations among FAAs, fatty acids, and volatile compounds in lamb from different breeds, and an integrative multivariate analysis identified strong associations among specific amino acids, unsaturated fatty acids, and key VOCs in lamb [11]. Together, these findings suggest that differences in non-volatile precursor composition may provide an important chemical basis for breed-related volatile flavor characteristics. The significant negative correlations of Ile and Lys with o-xylene may also reflect shared upstream metabolic or oxidative pathways, but the possibility of direct precursor–product conversion cannot be established from these statistical associations alone [40,41]. Previous studies have demonstrated that changes in phospholipids and other lipid components are closely associated with volatile compound formation in lamb [42,43]. However, the formation of individual volatile compounds is not governed by a single lipid degradation pathway. Instead, it involves multiple reactions and their interactions, including lipid oxidation, the Maillard reaction, and Strecker degradation [44].

Therefore, Tyr, C20:3n-6, and C22:4n-6 should be considered candidate flavor-associated compounds that co-vary with VOC profiles in unadjusted analyses, rather than confirmed direct precursors of specific VOCs. Similarly, Ile and Lys should be regarded as compounds showing robust statistical co-variation with o-xylene, but not as experimentally confirmed precursors. Overall, the three sheep breeds exhibited similar basic meat quality characteristics, whereas breed-related flavor differences were mainly reflected in selective variations in specific FAAs, FFAs, and VOCs with high rOAVs. Differences in non-volatile flavor-related compound composition may be associated with variation in VOC profiles, potentially through processes involving lipid oxidation and amino acid-related reactions. However, the precursor–VOC relationships identified in this study are primarily based on statistical associations. To verify true precursor–product relationships, future studies should employ controlled model heating systems combined with stable-isotope labelling to trace the formation of specific VOCs from defined precursors. In parallel, GC–O and aroma recombination/omission experiments would be valuable for evaluating the sensory relevance of the identified VOCs.

## 5. Conclusions

This study demonstrated that CLH, AH, and BDT exhibited generally similar basic meat quality characteristics but differed in meat color and the profiles of FAAs, FFAs, and VOCs. CLH and BDT showed higher Glu and total UAA contents than AH, whereas CLH had the highest total SAA content. BDT also exhibited relatively high Cys and C18:0 contents, while AH showed higher levels of C20:3n-6 and C22:4n-6. The rOAV analysis and odor attribute annotation further suggested that breed-related aroma differences were mainly associated with specific potentially aroma-active VOCs. However, no sensory or commercial validation was conducted in this study. Therefore, these findings are limited to the observed chemical differences, and their sensory relevance and potential commercial application require further validation through descriptive sensory analysis, cooked-meat VOC analysis, and larger-scale evaluation.

## Figures and Tables

**Figure 1:**
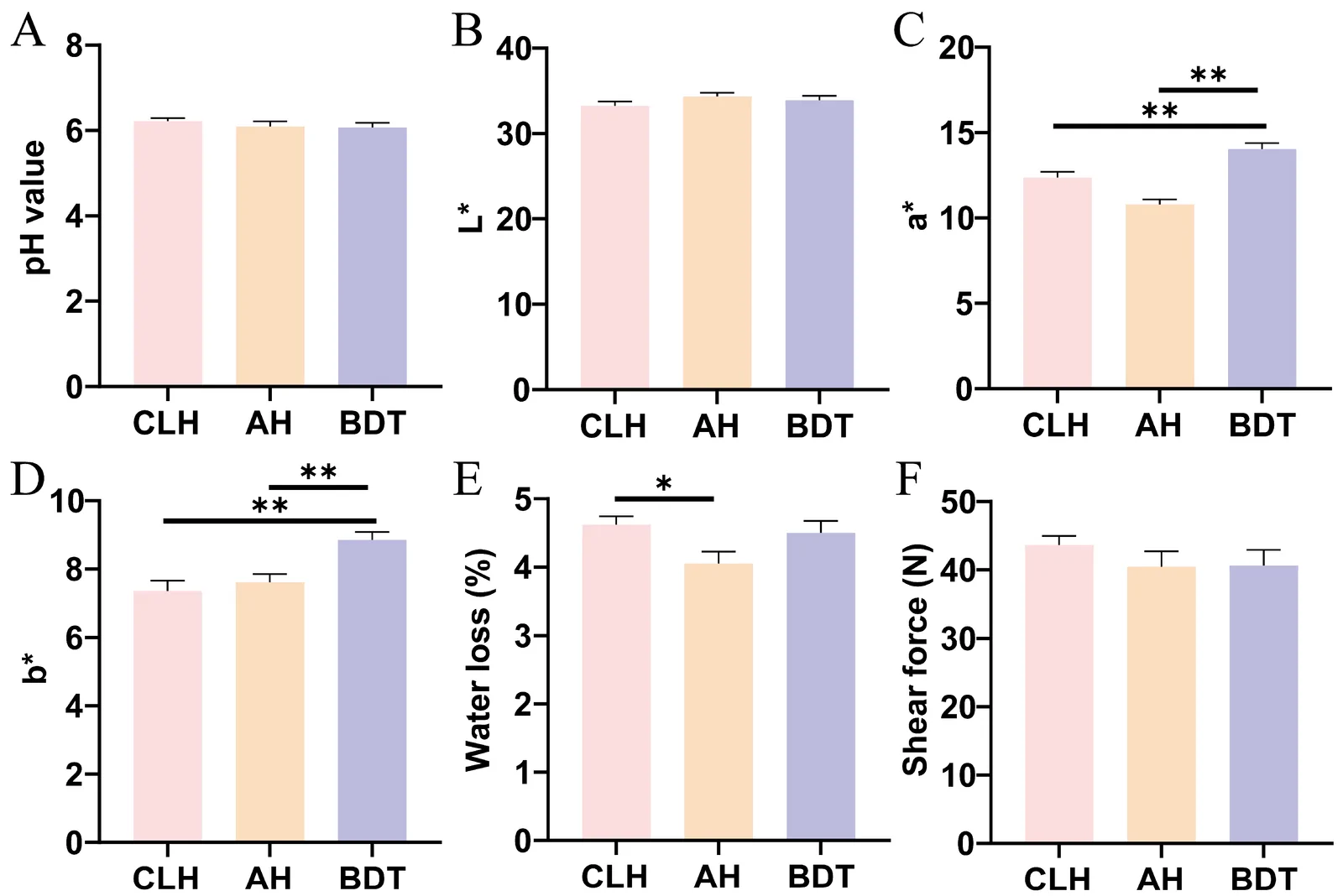
Meat quality characteristics of the longissimus thoracis muscle from different sheep breeds. (**A**) pH; (**B**) lightness (L*); (**C**) redness (a*); (**D**) yellowness (b*); (**E**) water loss rate; and (**F**) shear force. Data are presented as the mean ± SEM (n = 6 sheep per breed). Horizontal brackets indicate pairwise comparisons between the connected groups. Asterisks indicate statistically significant differences: *p* < 0.05 (*) and *p* < 0.01 (**). Panels without significance markers indicate no statistically significant differences among groups. CLH, Cele Black sheep; AH, Australian White × Hu crossbred sheep; BDT, Hotan sheep.

**Figure 2:**
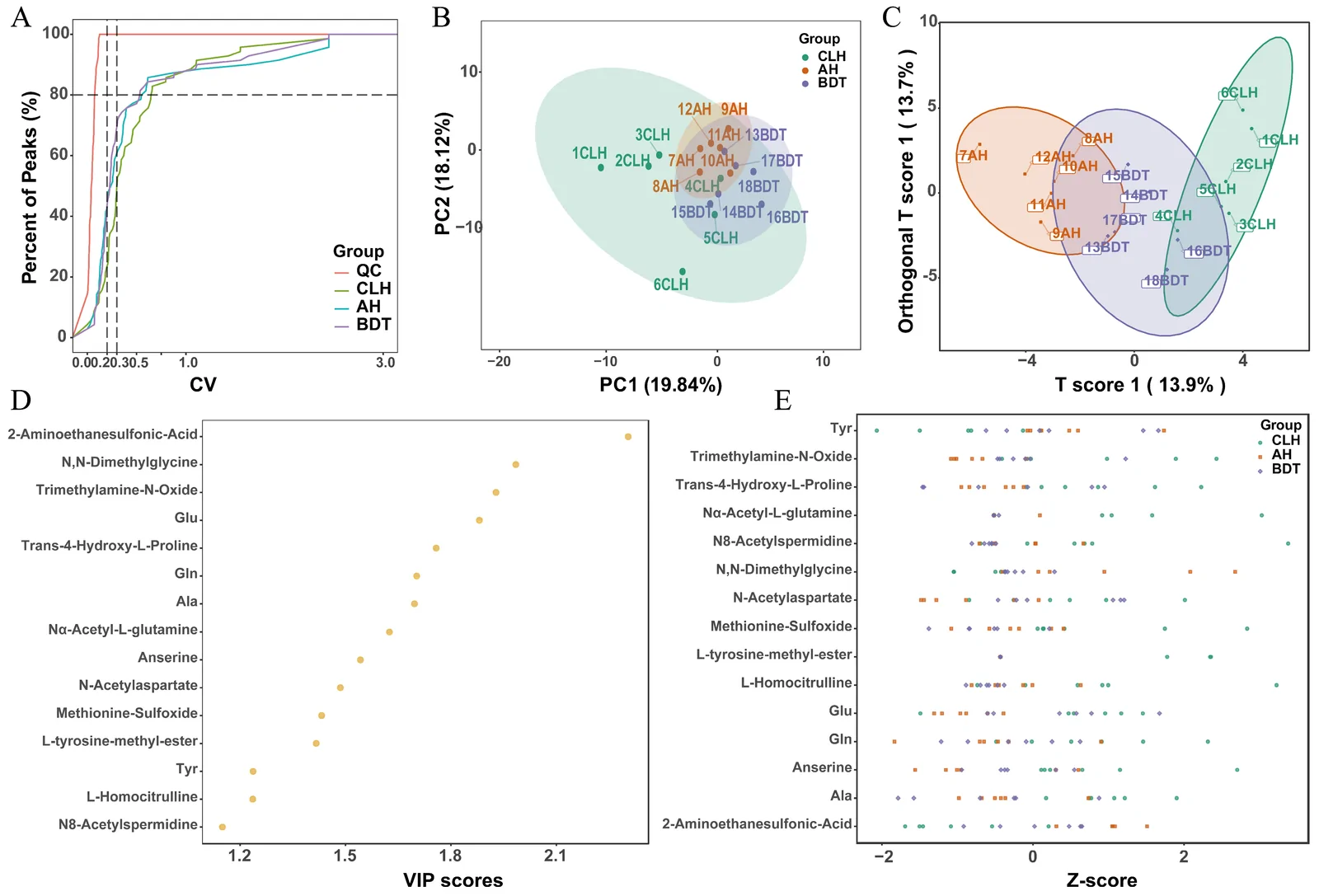
Free amino acid profiling and multivariate analysis of the *longissimus thoracis* muscle from different sheep breeds. (**A**) Distribution of coefficients of variation (CVs) for amino acids and related metabolites in quality control (QC) samples; (**B**) principal component analysis (PCA) score plot of CLH, AH, and BDT samples; (**C**) orthogonal partial least-squares discriminant analysis (OPLS-DA) score plot of CLH, AH, and BDT samples, where green, orange, and purple represent CLH, AH, and BDT, respectively; (**D**) variable importance in projection (VIP) scores of metabolites contributing to breed discrimination; and (**E**) Z-score visualization of representative differential metabolites among the three breeds.

**Figure 3:**
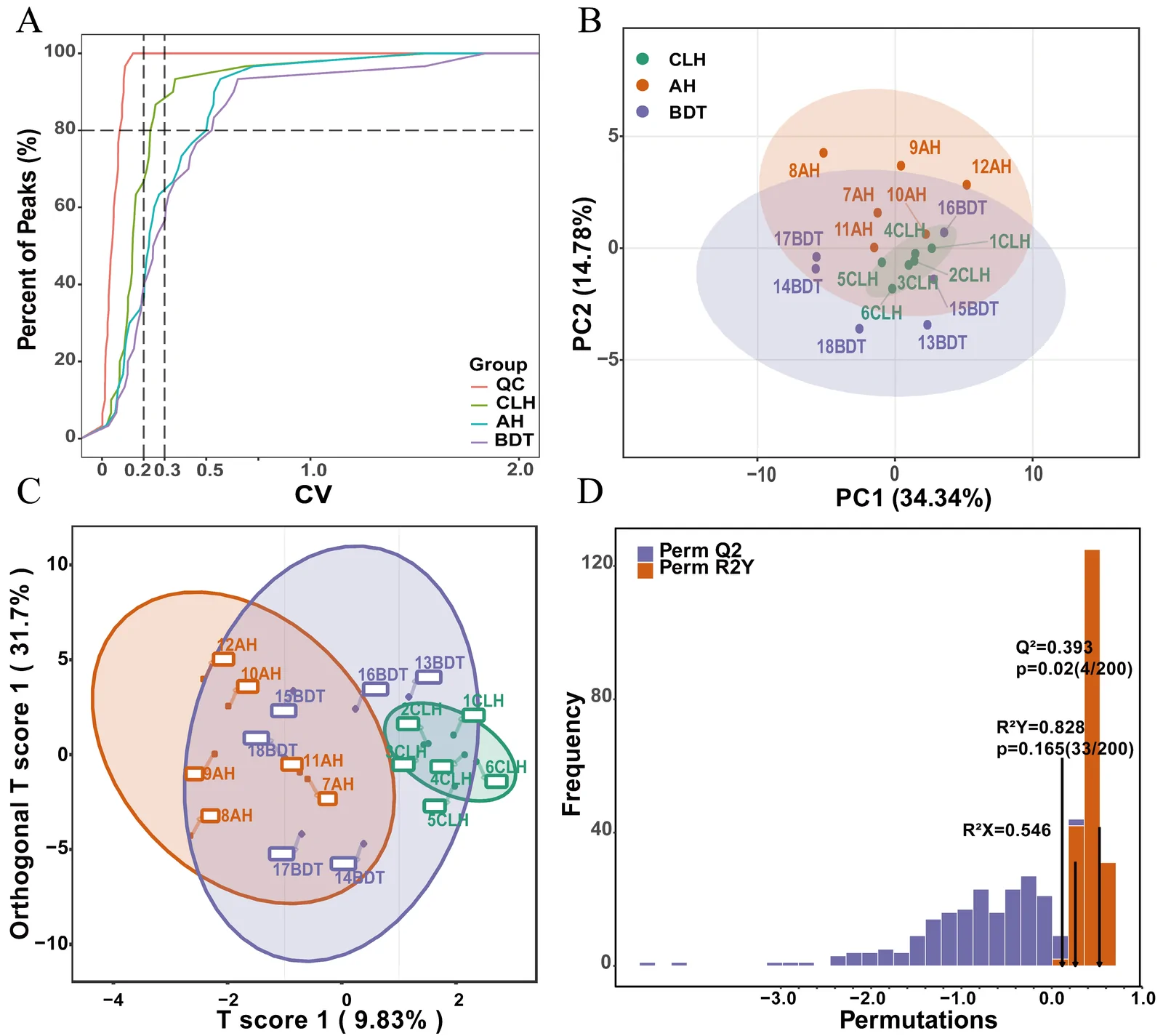
Free fatty acid profiling and multivariate analysis of the *longissimus thoracis* muscle from different sheep breeds. (**A**) Distribution of CVs for FFAs in QC samples; (**B**) PCA score plot of CLH, AH, and BDT samples; (**C**) OPLS-DA score plot of CLH, AH, and BDT; and (**D**) 200-permutation test of the OPLS-DA model.

**Figure 4:**
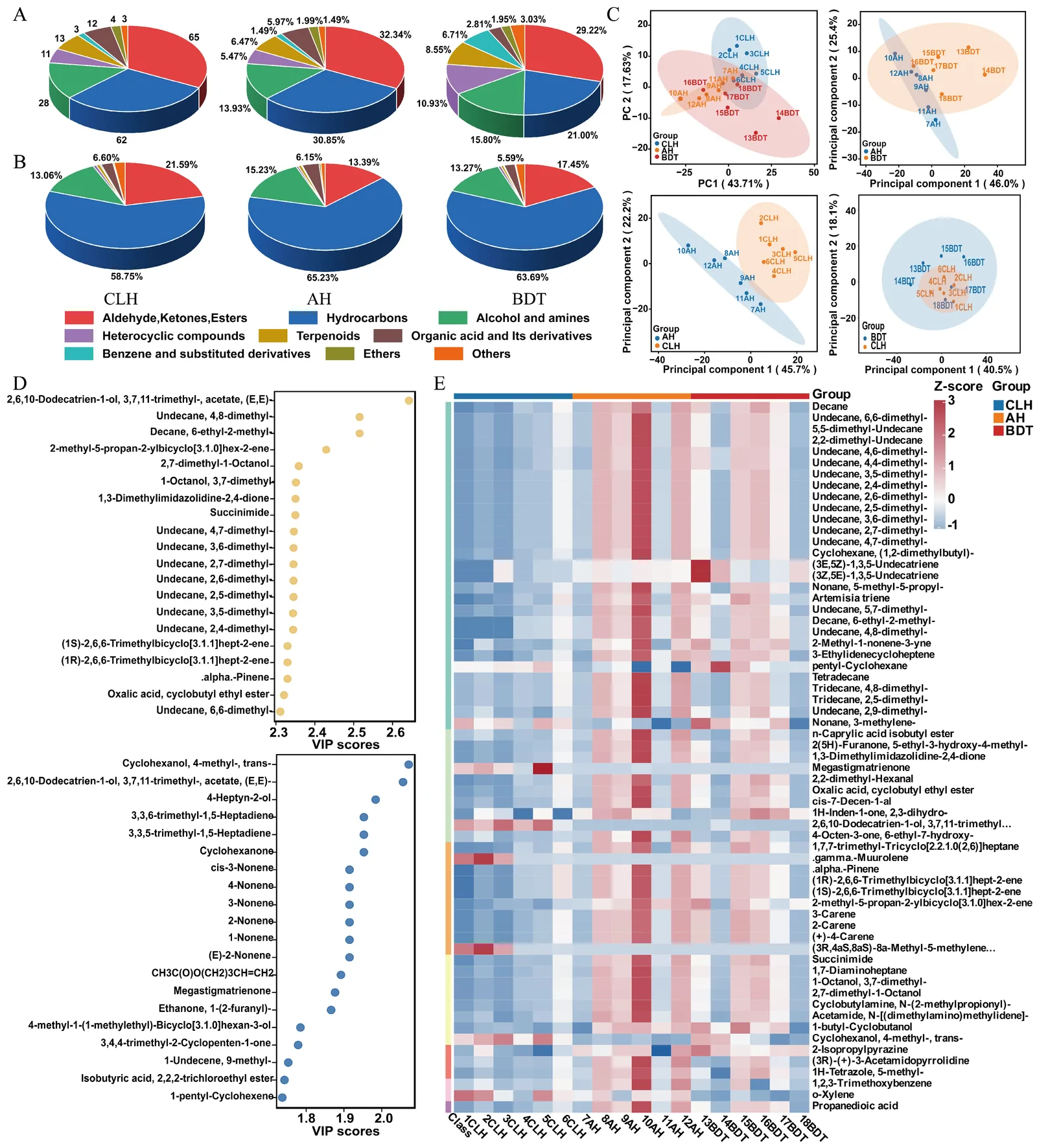
Volatile organic compound profiles and multivariate analysis of the *longissimus thoracis* muscle from different sheep breeds. (**A**) Distribution of the 201 annotated VOCs according to chemical class; (**B**) relative abundance of the major VOC classes in CLH, AH, and BDT; (**C**) multivariate analysis of VOC profiles, including PCA of CLH, AH, and BDT samples and pairwise OPLS-DA for AH vs. BDT, AH vs. CLH, and BDT vs. CLH; (**D**) VIP analysis for the overall comparison among CLH, AH, and BDT and for the AH vs. CLH comparison; and (**E**) hierarchical clustering analysis of representative differential VOCs based on their standardized relative abundances.

**Figure 5:**
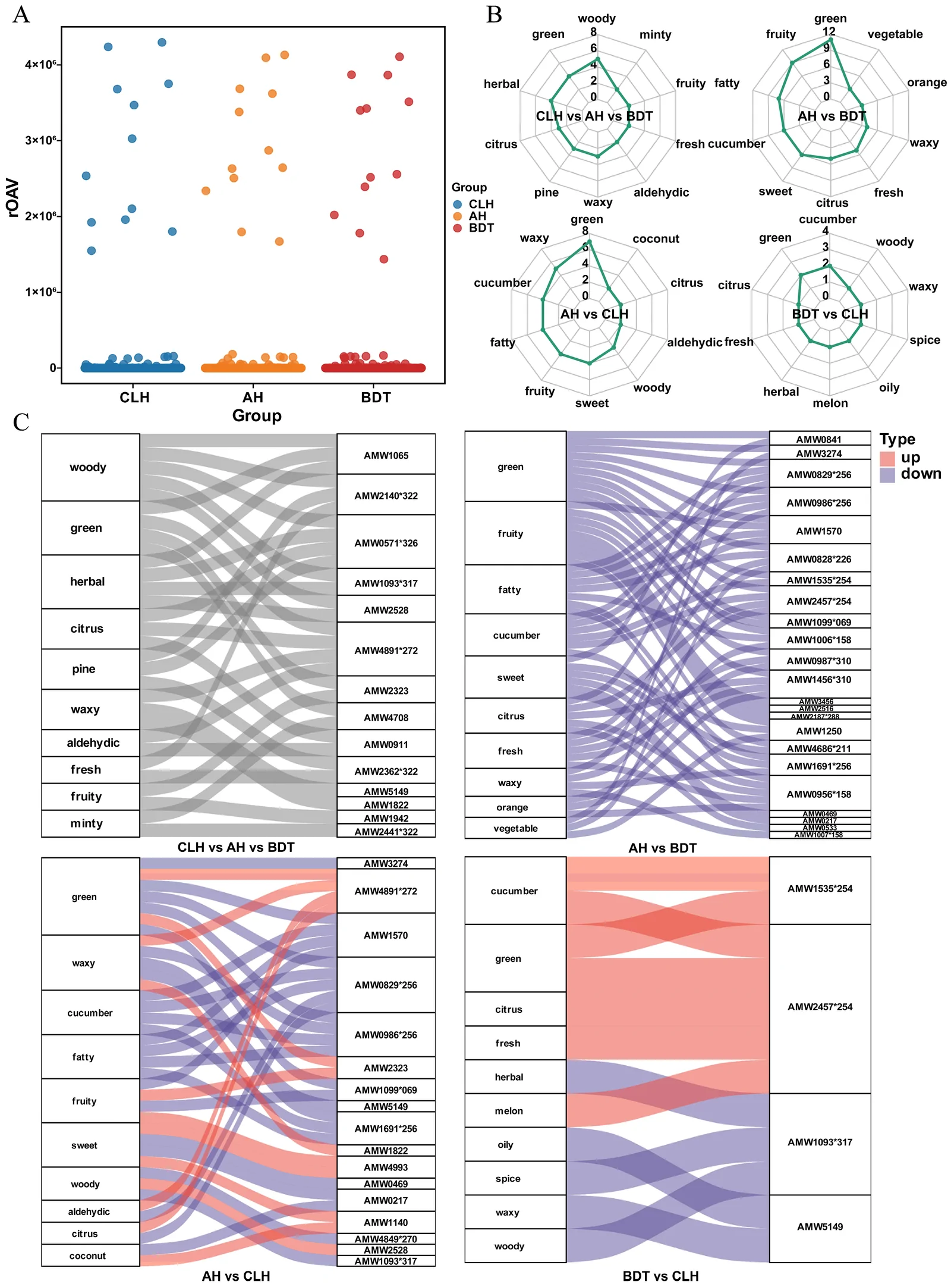
Potential key aroma-active compounds and odor attributes of differential VOCs in different sheep breeds. (**A**) rOAVs of potential aroma-active VOCs in CLH, AH, and BDT; compounds with rOAV ≥ 1 were considered to have potential contributions to the overall aroma profile. (**B**) Distribution of odor attributes associated with differential VOCs in the overall and pairwise comparisons. Numbers indicate the number of differential VOCs annotated to each odor attribute. (**C**) Sankey diagram illustrating the relationships among differential VOCs, their direction of change, and associated odor attributes. Asterisks (*) indicate that the compound is an isomer of other compounds with the same ID.

**Figure 6:**
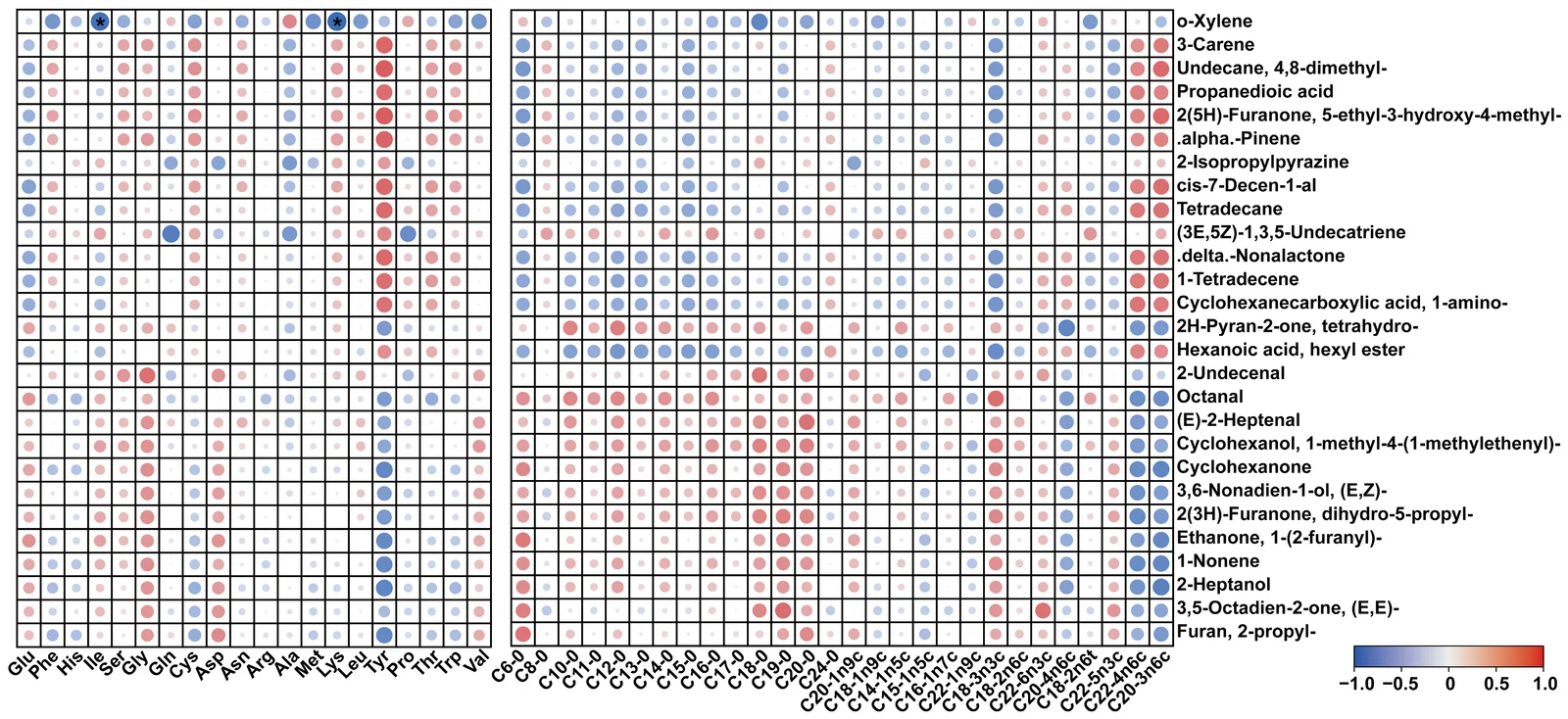
Associations between non-volatile flavor-related compounds and potential key VOCs in different sheep breeds. Spearman correlation analysis was performed among FAAs, FFAs, and potential key VOCs in CLH, AH, and BDT sheep. Only associations that remained significant after FDR correction are shown. Asterisks indicate FDR-adjusted *p* < 0.05 (*). Red indicates a positive correlation, whereas blue indicates a negative correlation. The color intensity reflects the strength of the correlation.

**Table 1:** Free amino acid contents in the *longissimus thoracis* muscle of different sheep breeds (μg/g).

Amino Acid	CLH	AH	BDT	*p*-Value
Glu	27.77 ± 2.00 ^A^	21.21 ± 0.66 ^B^	27.11 ± 1.62 ^A^	0.016
Phe	16.72 ± 1.47	18.32 ± 0.90	20.46 ± 1.64	0.189
His	33.20 ± 2.63	36.19 ± 4.06	38.00 ± 2.72	0.577
Ile	16.21 ± 0.76 ^AB^	14.75 ± 1.00 ^B^	19.52 ± 1.55 ^A^	0.029
Ser	36.19 ± 5.06	32.56 ± 2.46	40.87 ± 1.22	0.239
Gly	107.78 ± 11.83	90.84 ± 6.73	131.10 ± 12.43	0.053
Gln	832.98 ± 92.46 ^A^	544.30 ± 79.84 ^B^	598.34 ± 62.52 ^AB^	0.048
Cys	5.39 ± 0.64 ^B^	7.07 ± 0.58 ^AB^	8.96 ± 0.89 ^A^	0.011
Asp	5.05 ± 0.59	3.87 ± 0.20	4.22 ± 0.50	0.211
Asn	19.61 ± 5.28	20.27 ± 2.89	24.05 ± 2.35	0.670
Arg	83.37 ± 5.10	83.01 ± 7.07	90.50 ± 7.49	0.674
Ala	1050.50 ± 16.05 ^A^	958.46 ± 16.26 ^AB^	942.05 ± 27.28 ^B^	0.004
Met	6.19 ± 0.75	6.19 ± 0.35	7.22 ± 0.89	0.505
Lys	26.87 ± 2.42 ^B^	31.21 ± 1.98 ^AB^	38.95 ± 3.49 ^A^	0.020
Leu	28.08 ± 2.38	29.90 ± 1.16	33.18 ± 1.88	0.184
Tyr	18.88 ± 2.18 ^B^	27.67 ± 1.80 ^A^	27.27 ± 2.51 ^A^	0.020
Pro	42.70 ± 6.44	33.47 ± 2.02	32.93 ± 2.60	0.211
Thr	27.60 ± 3.69	26.05 ± 2.84	28.77 ± 2.01	0.807
Trp	8.22 ± 0.61	8.47 ± 0.43	9.39 ± 0.69	0.360
Val	29.75 ± 2.22	29.40 ± 1.06	31.77 ± 1.41	0.554
SAA	1264.77 ± 18.08 ^A^	1141.38 ± 23.59 ^B^	1175.73 ± 36.20 ^B^	0.016
UAA	32.81 ± 2.07 ^A^	25.08 ± 0.64 ^B^	31.32 ± 1.91 ^A^	0.012
EAA	192.82 ± 13.83	200.48 ± 10.09	227.27 ± 13.36	0.160

Different superscript letters within the same row indicate significant differences among breeds (*p* < 0.05). Glu, glutamic acid; Phe, phenylalanine; His, histidine; Ile, isoleucine; Ser, serine; Gly, glycine; Gln, glutamine; Cys, cysteine; Asp, aspartic acid; Asn, asparagine; Arg, arginine; Ala, alanine; Met, methionine; Lys, lysine; Leu, leucine; Tyr, tyrosine; Pro, proline; Thr, threonine; Trp, tryptophan; Val, valine. SAA, sweet amino acids, including Gly, Ala, Ser, Thr, and Pro; UAA, umami amino acids, including Glu and Asp; EAA, essential amino acids, including His, Ile, Leu, Lys, Met, Phe, Thr, Trp, and Val.

**Table 2:** Free fatty acid contents in the *longissimus thoracis* muscle of different sheep breeds (μg/g).

Fatty Acid	CLH	AH	BDT	*p*-Value
C6:0	0.39 ± 0.03 ^A^	0.27 ± 0.04 ^B^	0.27 ± 0.05 ^B^	0.048
C8:0	0.35 ± 0.09	0.59 ± 0.17	0.38 ± 0.03	0.316
C10:0	2.10 ± 0.29	2.50 ± 0.55	2.95 ± 0.76	0.580
C11:0	0.07 ± 0.01	0.09 ± 0.01	0.08 ± 0.01	0.566
C12:0	1.36 ± 0.12	1.42 ± 0.22	1.79 ± 0.48	0.582
C13:0	0.64 ± 0.01	0.64 ± 0.02	0.65 ± 0.03	0.890
C14:0	11.21 ± 1.05	12.65 ± 2.22	16.05 ± 3.88	0.436
C15:0	2.91 ± 0.15	2.83 ± 0.32	2.97 ± 0.23	0.972
C16:0	125.15 ± 8.29	126.76 ± 12.49	148.28 ± 16.27	0.384
C17:0	5.06 ± 0.29	5.31 ± 0.49	5.62 ± 0.79	0.785
C18:0	41.10 ± 2.45 ^B^	36.37 ± 3.17 ^B^	63.72 ± 4.87 ^A^	<0.001
C19:0	0.81 ± 0.01 ^A^	0.78 ± 0.01 ^B^	0.80 ± 0.01 ^A^	0.008
C20:0	0.74 ± 0.07	0.65 ± 0.03	0.79 ± 0.04	0.166
C24:0	0.86 ± 0.12	0.97 ± 0.21	0.91 ± 0.11	0.876
SFA	192.73 ± 11.64	191.83 ± 14.37	245.29 ± 23.80	0.075
C14:1n-5c	1.26 ± 0.05	1.65 ± 0.34	1.52 ± 0.19	0.497
C15:1n-5c	10.47 ± 0.45	11.02 ± 0.32	10.65 ± 0.53	0.683
C16:1n-7c	15.38 ± 0.96	19.88 ± 4.61	17.64 ± 3.26	0.638
C18:1n-9c	255.94 ± 14.37	259.31 ± 26.47	284.02 ± 28.22	0.668
C20:1n-9c	1.10 ± 0.04	1.04 ± 0.04	1.06 ± 0.03	0.531
C22:1n-9c	1.60 ± 0.16	1.63 ± 0.15	1.58 ± 0.13	0.965
MUFA	285.95 ± 14.48	294.52 ± 30.42	316.47 ± 31.77	0.710
C18:2n-6c	68.30 ± 5.55	72.44 ± 3.51	75.46 ± 9.44	0.750
C18:2n-6t	16.14 ± 0.56	16.51 ± 1.39	18.47 ± 1.44	0.365
C18:3n-3c	5.41 ± 0.26	4.73 ± 0.38	5.40 ± 0.37	0.311
C20:3n-6c	10.55 ± 0.62 ^B^	14.74 ± 1.08 ^A^	12.96 ± 0.85 ^AB^	0.013
C20:4n-6c	76.07 ± 3.87	79.68 ± 5.52	71.70 ± 2.19	0.194
C22:4n-6c	7.31 ± 0.25 ^B^	9.33 ± 0.42 ^A^	7.29 ± 0.45 ^B^	0.002
C22:5n-3c	7.93 ± 0.48	7.00 ± 0.30	7.14 ± 0.72	0.432
C22:6n-3c	2.05 ± 0.18	1.77 ± 0.15	1.88 ± 0.40	0.769
PUFA	193.75 ± 9.76	206.20 ± 6.76	200.29 ± 8.31	0.334

Different superscript letters within the same row indicate significant differences among breeds (*p* < 0.05). SFA, saturated fatty acids; MUFA, monounsaturated fatty acids; PUFA, polyunsaturated fatty acids. SFA, MUFA, and PUFA represent the sums of the corresponding individual fatty acids.

## Data Availability

The original contributions presented in this study are included in the article/Supplementary Material. Further inquiries can be directed to the corresponding author.

## Supplementary Materials

The following supporting information can be downloaded at: https://www.mdpi.com/article/10.3390/foods15213945/s1, Table S1: Dietary composition and nutritional level; Table S2: Free amino acid profiles of different sheep breeds; Table S3: Compound-specific LC–MS/MS MRM transitions, retention times, and identification evidence for amino acids and related metabolites; Table S4: Identification, abundance, and statistical summary of 201 VOCs; Figure S1: Multivariate model validation and differential analysis of free amino acids among sheep breeds; Figure S2: Quality assessment and OPLS-DA of volatile organic compound profiles among sheep breeds.
