# Supplementary material for: Comparative Profiling of Free Amino Acids, Free Fatty Acids, and Volatile Organic Compounds in the Longissimus Thoracis Muscle of Three Sheep Breed Groups
Source: Foods. 2026 Nov 3;15(21):3945. doi: 10.3390/foods15213945 (PMC13763103; doi:10.3390/foods15213945)
Supplement: Supplementary file 1 [file foods-15-03945-s001.zip › foods-4628206-supplementary.pdf]

Supplementary Materials

Comparative Profiling of Free Amino Acids, Free Fatty Acids, and Volatile Organic Compounds in the Longissimus Thoracis Muscle of Three Sheep Breed Groups

Table S1 Dietary composition and nutritional level

**Ingredient**	**g/kg**	**Nutrient composition**	（%）
Corn	593	Crude protein (CP)	15.69
Cottonseed meal	60	Neutral detergent fiber (NDF)	27.51
Wheat flour	120	Acid detergent fiber (ADF)	14.19
Soybean meal (43% CP)	120	Crude ash	8.05
Safflower meal	50	Calcium (Ca)	0.91
Limestone	20	Phosphorus (P)	0.41
Premix	15	Sodium chloride	1.00
Palm fat powder	8	Moisture	12.00
Salt	10	ME (MJ/kg)	11.43
Sodium bicarbonate	4		
Total	1000		

ME was calculated based on the ingredient composition and reference feed values; the remaining nutrient contents were determined analytically. The premix was included at 15 g/kg diet and provided, per kilogram of premix, 267,000 IU vitamin A, 80,000 IU vitamin D_3, 733 mg vitamin E, 3,733 mg Fe, 4,800 mg Zn, 867 mg Cu, 1,200 mg Mn, 21.3 mg Se, 20 mg I, and 26.7 mg Co.

Table S2 Free amino acid profiles of different sheep breeds

Amino acid	CLH	AH	BDT	P value
Nα-Acetyl-L-Arginine	162.62±17.75	149.40±11.31	155.65±8.25	0.799
L-Homocitrulline	23706.20±4681.54^{A}	6692.95±1002.41^{B}	4737.62±761.52^{B}	0.028
Homo-Arg	5228.11±338.07	5202.80±295.62	5285.56±280.11	0.135
N6-Acetyl-L-Lysine	1197.06±136.42	1096.93±104.89	1275.55±116.42	0.296
Beta-Alanine	190.45±29.88	168.88±19.43	177.65±21.73	0.548
3-N-Methyl-L-Histidine	10198.22±1085.43	12480.76±1224.35	15109.62±1770.04	0.066
N-Acetylneuraminic-Acid	370.92±46.20	414.11±31.85	393.61±41.42	0.474
N-Acetylaspartate	121.62±11.42^{B}	191.54±19.18^{A}	183.22±15.75^{A}	0.02
L-Citrulline	118.51±9.68	104.55±12.13	127.42±13.80	0.115
kynurenine	1.21±0.15	1.08±0.11	1.31±0.12	0.443
γ-Aminobutyric-Acid	14.17±1.48	15.50±1.52	17.23±1.86	0.481
α-Aminoadipic-acid	3.35±0.43	3.03±0.32	3.62±0.39	0.546
2-Aminobutyric-acid	3.61±0.44	4.20±0.38	4.86±0.61	0.136
5-Aminovaleric-Acid	1.02±0.10	0.94±0.08	1.05±0.12	0.828
Creatine	12465.77±1043.55	13213.89±1055.20	14386.52±1202.31	0.429
Anserine	13425.31±1056.24^{B}	15440.21±1132.18^{AB}	17762.66±1321.04^{A}	0.022
Methionine-Sulfoxide	112.36±8.14^{B}	143.25±10.55^{AB}	174.20±13.21^{A}	0.012
Phosphorylethanolamine	134.28±10.11	151.38±12.52	159.42±13.80	0.182
(5-L-Glutamyl)-L-Alanine	32.16±3.10	29.84±2.86	34.57±3.24	0.633
Trimethylamine-N-Oxide	26.19±2.08^{B}	32.12±2.42^{AB}	41.35±3.16^{A}	0.013
Urea	107.34±9.24^{B}	126.57±10.11^{AB}	156.34±12.25^{A}	0.024
argininosuccinic-acid	4.23±0.35	4.01±0.32	4.72±0.40	0.103
3-Aminoisobutanoic-Acid	2.14±0.21	2.58±0.23	3.11±0.30	0.074
D-Homocysteine	0.44±0.05	0.41±0.04	0.46±0.05	0.961
Glutathione-Oxidized	8.62±0.72	8.41±0.66	8.91±0.75	0.788
Glycyl-L-Proline	23.64±2.11	25.31±2.34	27.42±2.52	0.22
L-Carnosine	11542.13±965.21	12411.65±1022.40	13255.42±1118.36	0.609
S-(5-Adenosyl)-L-Homocysteine	1.15±0.12	1.09±0.10	1.23±0.13	0.554
1-Methylhistidine	401.28±32.15^{B}	488.53±38.21^{AB}	612.84±45.30^{A}	0.027
Trans-4-Hydroxy-L-Proline	56.32±4.80^{B}	70.14±5.31^{AB}	86.23±6.42^{A}	0.022
Succinic-Acid	15.23±1.44	14.88±1.32	16.02±1.50	0.841
N8-Acetylspermidine	2.31±0.22^{B}	2.85±0.25^{AB}	3.41±0.31^{A}	0.041

Table S3 Compound-specific LC–MS/MS MRM transitions, retention times, and identification evidence for amino acids and related metabolites.

No.	Analyte	CAS No.	Mode	Ion species	Q1	Q3	MRM transition	RT (min)	Standard†	Criteria‡
1	L-Homocitrulline	1190-49-4	ESI+	[M+H]+	190.1	84.08	190.1 → 84.08	8.332	ND†	MRM+RT‡
2	Glutathione Oxidized	121-24-4	ESI+	[M+H]+	613.152	355.7	613.152 → 355.7	10.879	ND†	MRM+RT‡
3	N-Acetylneuraminic Acid	131-48-6	ESI+	[M+H]+	310.11	274.09	310.11 → 274.09	8.866	ND†	MRM+RT‡
4	N8-Acetylspermidine	13431-24-8	ESI+	[M+H]+	190.18	173.15	190.18 → 173.15	8.332	ND†	MRM+RT‡
5	Ethanolamine	141-43-5	ESI+	[M+H]+	62	45	62 → 45	3.292	ND†	MRM+RT‡
6	3-Aminoisobutanoic Acid	144-90-1	ESI+	[M+H]+	104.07	86.05	104.07 → 86.05	4.44	ND†	MRM+RT‡
7	L-Proline	147-85-3	ESI+	[M+H]+	116.1	70.1	116.1 → 70.1	4.072	ND†	MRM+RT‡
8	Phosphorylethanolamine	1071-23-4	ESI−	[M-H]−	140.1	79	140.1 → 79	9.48	ND†	MRM+RT‡
9	2-Aminobutyric acid	1492-24-6	ESI+	[M+H]+	104.1	58.14	104.1 → 58.14	5.02	ND†	MRM+RT‡
10	Nα-Acetyl-L-Arginine	155-84-0	ESI+	[M+H]+	217.1	158	217.1 → 158	7.2	ND†	MRM+RT‡
11	Homo-L-arginine	156-86-5	ESI+	[M+H]+	189	144	189 → 144	9.52	ND†	MRM+RT‡
12	N-Isovaleroylglycine	16284-60-9	ESI+	[M+H]+	160.1	76	160.1 → 76	1.107	ND†	MRM+RT‡
13	3-Hydroxyhippuric Acid	1637-75-8	ESI+	[M+H]+	196.05	121	196.05 → 121	1.417	ND†	MRM+RT‡
14	N-Propionylglycine	21709-	ESI	[M+	132	76	132 →	1.60	ND†	MRM+

		90-0	+	H]+			76	1		RT‡
15	Argininosuccinic acid	2387-71-5	ESI +	[M+H]+	291.5	176.1	291.5 → 176.1	10.582	ND†	MRM+ RT‡
16	Nα-Acetyl-L-glutamine	2490-97-3	ESI +	[M+H]+	189.1	84.04	189.1 → 84.04	3.361	ND†	MRM+ RT‡
17	2-Aminoethanesulfonic Acid	107-35-7	ESI +	[M+H]+	126.02	44	126.02 → 44	4.333	ND†	MRM+ RT‡
18	4-Acetamidobutyric Acid	3025-96-5	ESI +	[M+H]+	146.074	128	146.074 → 128	1.041	ND†	MRM+ RT‡
19	L-Carnosine	305-84-0	ESI +	[M+H]+	227.2	156	227.2 → 156	9.991	ND†	MRM+ RT‡
20	L-Theanine	3081-61-6	ESI +	[M+H]+	175	158	175 → 158	4.771	ND†	MRM+ RT‡
21	L-Pipecolic Acid	3105-95-1	ESI +	[M+H]+	130	56.05	130 → 56.05	4.305	ND†	MRM+ RT‡
22	1-Methylhistidine	332-80-9	ESI +	[M+H]+	170.1	109.2	170.1 → 109.2	8.395	ND†	MRM+ RT‡
23	Kynurenine	343-65-7	ESI +	[M+H]+	209.1	146	209.1 → 146	2.742	ND†	MRM+ RT‡
24	3-N-Methyl-L-Histidine	368-16-1	ESI +	[M+H]+	170	109.07	170 → 109.07	9.587	ND†	MRM+ RT‡
25	L-Citrulline	372-75-8	ESI +	[M+H]+	176.1	70	176.1 → 70	8.646	ND†	MRM+ RT‡
26	Beta-Alanine	107-95-9	ESI +	[M+H]+	90.2	30	90.2 → 30	6.576	ND†	MRM+ RT‡
27	D-Homocysteine	454-29-5	ESI +	[M+H]+	193	88	193 → 88	7.559	ND†	MRM+ RT‡
28	Kynurenic Acid	492-27-3	ESI +	[M+H]+	190.043	89	190.043 → 89	1.982	ND†	MRM+ RT‡
29	5-Hydroxy-Tryptamine	50-67-9	ESI +	[M+H]+	177.1	160	177.1 → 160	4.808	ND†	MRM+ RT‡
30	Trans-4-Hydroxy-L-Proline	51-35-4	ESI +	[M+H]+	132.058	86	132.058 → 86	6.566	ND†	MRM+ RT‡
31	L-Cysteine	52-90-4	ESI	[M+	179	116	179 →	7.60	ND†	MRM+

			+	H]+			116	4		RT‡
32	α-Aminoadipic acid	542-32-5	ESI +	[M+H]+	162.07	98.06	162.07 → 98.06	8.251	ND†	MRM+ RT‡
33	γ-Aminobutyric Acid	1956-12-2	ESI +	[M+H]+	104.06	69	104.06 → 69	4.261	ND†	MRM+ RT‡
34	Glycine	56-40-6	ESI +	[M+H]+	76.03	30	76.03 → 30	7.604	ND†	MRM+ RT‡
35	L-Alanine	56-41-7	ESI +	[M+H]+	90.05	44.05	90.05 → 44.05	6.616	ND†	MRM+ RT‡
36	L-Serine	56-45-1	ESI +	[M+H]+	106.04	70	106.04 → 70	8.202	ND†	MRM+ RT‡
37	L-Aspartate	56-84-8	ESI +	[M+H]+	134.04	116	134.04 → 116	9.376	ND†	MRM+ RT‡
38	L-Glutamine	56-85-9	ESI +	[M+H]+	147.069	84	147.069 → 84	8.108	ND†	MRM+ RT‡
39	L-Glutamic acid	56-86-0	ESI +	[M+H]+	148.06	84	148.06 → 84	8.776	ND†	MRM+ RT‡
40	L-Lysine	56-87-1	ESI +	[M+H]+	147.069	56	147.069 → 56	9.955	ND†	MRM+ RT‡
41	L-tyrosine methyl ester	1080-06-4	ESI +	[M+H]+	196.2	179.1	196.2 → 179.1	1.217	ND†	MRM+ RT‡
42	L-Cystathionine	56-88-2	ESI +	[M+H]+	223.07	134	223.07 → 134	10.431	ND†	MRM+ RT‡
43	Creatine	57-00-1	ESI +	[M+H]+	132.1	44	132.1 → 44	6.646	ND†	MRM+ RT‡
44	Urea	57-13-6	ESI +	[M+H]+	61	44	61 → 44	1.293	ND†	MRM+ RT‡
45	Anserine	584-85-0	ESI +	[M+H]+	241	170	241 → 170	9.885	ND†	MRM+ RT‡
46	(5-L-Glutamyl)-L-Alanine	5875-41-2	ESI +	[M+H]+	219.09	202	219.09 → 202	9.31	ND†	MRM+ RT‡
47	L-Tyrosine	60-18-4	ESI +	[M+H]+	182.08	91.05	182.08 → 91.05	4.305	ND†	MRM+ RT‡
48	Succinic Acid	110-15-	ESI	[M-	117.	73	117.03	1.13	ND†	MRM+

		6	−	H]−	03		→ 73	7		RT‡
49	L-Leucine	61-90-5	ESI+	[M+H]+	132.1	44.04	132.1 → 44.04	2.681	ND†	MRM+ RT‡
50	Methionine Sulfoxide	62697-73-8	ESI+	[M+H]+	166	74	166 → 74	8.057	ND†	MRM+ RT‡
51	L-Methionine	63-68-3	ESI+	[M+H]+	150.05	104	150.05 → 104	3.343	ND†	MRM+ RT‡
52	L-Phenylalanine	63-91-2	ESI+	[M+H]+	166.1	103.05	166.1 → 103.05	2.607	ND†	MRM+ RT‡
53	5-Aminovaleric Acid	660-88-8	ESI+	[M+H]+	118.2	55	118.2 → 55	2.964	ND†	MRM+ RT‡
54	N6-Acetyl-L-Lysine	692-04-6	ESI+	[M+H]+	189.1	126	189.1 → 126	7.055	ND†	MRM+ RT‡
55	L-Ornithine	70-26-8	ESI+	[M+H]+	133.09	70	133.09 → 70	10.2	ND†	MRM+ RT‡
56	Glycyl-L-Proline	704-15-4	ESI+	[M+H]+	173.1	116.071	173.1 → 116.071	8.316	ND†	MRM+ RT‡
57	N,N-Dimethylglycine	1118-68-9	ESI+	[M+H]+	104	58	104 → 58	3.844	ND†	MRM+ RT‡
58	L-Asparagine Anhydrous	70-47-3	ESI+	[M+H]+	133.06	74	133.06 → 74	8.3	ND†	MRM+ RT‡
59	L-Histidine	71-00-1	ESI+	[M+H]+	156.07	110.07	156.07 → 110.07	9.39	ND†	MRM+ RT‡
60	L-Valine	72-18-4	ESI+	[M+H]+	118.1	55.05	118.1 → 55.05	3.827	ND†	MRM+ RT‡
61	L-Threonine	72-19-5	ESI+	[M+H]+	120.06	74	120.06 → 74	7.146	ND†	MRM+ RT‡
62	L-Tryptophan	73-22-3	ESI+	[M+H]+	205.1	118.06	205.1 → 118.06	2.694	ND†	MRM+ RT‡
63	L-Isoleucine	73-32-5	ESI+	[M+H]+	132.1	86.1	132.1 → 86.1	2.949	ND†	MRM+ RT‡
64	3-Chloro-L-Tyrosine	7423-9	ESI	[M+	216.	170.	216.1	3.15	ND†	MRM+

		3-0	+	H]+	1	1	→ 170.1			RT‡
65	L-Arginine	74-79-3	ESI+	[M+H]+	175.1	70.06	175.1 → 70.06	9.711	ND†	MRM+ RT‡
66	N-Glycyl-L-Leucine	869-19-2	ESI+	[M+H]+	189.1	86	189.1 → 86	4.591	ND†	MRM+ RT‡
67	Trimethylamine N-Oxide	1184-78-7	ESI+	[M+H]+	76	42.1	76 → 42.1	2.11	ND†	MRM+ RT‡
68	D-Alanyl-D-Alanine	923-16-0	ESI+	[M+H]+	161.09	90	161.09 → 90	7.796	ND†	MRM+ RT‡
69	S-(5-Adenosyl)-L-Homocysteine	979-92-0	ESI+	[M+H]+	385	88	385 → 88	8.646	ND†	MRM+ RT‡
70	N-Acetylaspartate	997-55-7	ESI+	[M+H]+	176	134	176 → 134	2.85	ND†	MRM+ RT‡

Table S4 Identification, abundance, and statistical summary of 201 VOCs

No.	Index	Quantitative ion	Qualitative ion	Molecular weight (Da)	Compounds	CAS	NIST_RI	Match factor	CLH	AH	BDT	P-value
1	AMW0535	85	98	142.172	Decane	124-18-5	1000	81.7	0.011	0.019	0.017	0.020
2	AMW1413	136	93	136.125	1,7,7-trimethyl-Tricyclo[2.2.1.0(2,6)]heptane	508-32-7	925	77.26	0.001	0.002	0.002	0.045
3	AMW2323	127	56	200.178	n-Caprylic acid isobutyl ester	5461-06-3	1348	73.08	0.002	0.004	0.002	0.048
4	AMW0121	68	96	156.115	5-Methyl-4-hexene-1-yl acetate	1000426-93-8	1108	81.37	0.171	0.103	0.213	0.131
5	AMW1942	113	142	142.063	2(5H)-Furanone, 5-ethyl-3-hydroxy-4-methyl-	698-10-2	1196	78.08	0.035	0.064	0.052	0.023
6	AMW0041	44	42	163.067	Carbomethoxy diethylamino sulfide	1000252-47-7	1110	58.51	0.166	0.106	0.205	0.166
7	AMW3381	100	99	128.059	1,3-Dimethylimidazolidine-2,4-dione	24039-08-5	1193	76.38	0.003	0.006	0.005	0.025
8	AMW2187*288	123	81	198.162	Menthyl acetate	89-48-5	1295	83.18	0.002	0.001	0.002	0.183
9	AMW3171*288	123	81	198.162	(-)-Neomenthyl acetate	146502-80-9	1277	83.18	0.002	0.001	0.002	0.183
10	AMW0889*288	123	81	198.162	(1.alpha.,2.beta.,5.beta.)-5-methyl-2-(1-methylethyl)-Cyclohexanol, acetate	20777-45-1	1305	83.18	0.002	0.001	0.002	0.183
11	AMW3227	105	119	190.136	Megastigmatrienone	38818-55-2	1473	71.9	0.014	0.000	7.9E-05	0.032
12	AMW0775*126	99	43	184.219	Undecane, 6,6-dimethyl-	17312-76-4	1202	82.72	0.045	0.085	0.068	0.023
13	AMW0773*126	99	43	184.219	5,5-dimethyl-Undecane	17312-73-1	1204	82.72	0.045	0.085	0.068	0.023
14	AMW0770*126	99	43	184.219	2,2-dimethyl-Undecane	17312-64-0	1214	82.72	0.045	0.085	0.068	0.023
15	AMW0777*369	84	71	184.219	Undecane, 4,6-dimethyl-	17312-82-2	1196	85.79	0.056	0.099	0.086	0.023
16	AMW0772*369	84	71	184.219	Undecane, 4,4-dimethyl-	17312-68-4	1216	85.79	0.056	0.099	0.086	0.023
17	AMW2860	70	56	99.032	Succinimide	123-56-8	1187	76.73	0.003	0.005	0.004	0.020
18	AMW0776*119	57	70	184.219	Undecane, 3,5-dimethyl-	17312-81-1	1207	81.79	0.395	0.810	0.633	0.022
19	AMW2390*119	57	70	184.219	Undecane, 2,4-dimethyl-	17312-80-0	1208	81.79	0.395	0.810	0.633	0.022
20	AMW0754*119	57	70	184.219	Undecane, 2,6-dimethyl-	17301-23-4	1210	80.51	0.390	0.806	0.628	0.022
21	AMW2426*119	57	70	184.219	Undecane, 2,5-dimethyl-	17301-22-3	1210	80.51	0.390	0.806	0.628	0.022
22	AMW2557*119	57	70	184.219	Undecane, 3,6-dimethyl-	17301-28-9	1210	80.51	0.390	0.806	0.628	0.022
23	AMW2705*119	57	70	184.219	Undecane, 2,7-dimethyl-	17301-24-5	1216	80.51	0.390	0.806	0.628	0.022
24	AMW0758*119	57	70	184.219	Undecane, 4,7-dimethyl-	17301-32-5	1209	82.2	0.393	0.809	0.631	0.022
25	AMW4896	56	44	130.147	1,7-Diaminoheptane	646-19-5	1197	78.53	0.076	0.143	0.117	0.025
26	AMW4898	55	85	168.188	Cyclohexane, (1,2-dimethylbutyl)-	61142-37-8	1200	80.45	0.055	0.101	0.082	0.028
27	AMW1093*317	147	161	204.188	.gamma.-Muurolene	30021-74-0	1477	77.38	0.005	8.5E-05	8.5E-05	0.031
28	AMW0246	83	69	130.136	2-ethyl-4-methyl-1-Pentanol	106-67-2	955	76.57	0.061	0.032	0.065	0.232
29	AMW2654*263	80	79	150.141	(3E,5Z)-1,3,5-Undecatriene	51447-08-6	1174	72.99	0.001	0.001	0.001	0.049
30	AMW4481*263	80	79	150.141	(3Z,5E)-1,3,5-Undecatriene	19883-27-3	1182	72.99	0.001	0.001	0.001	0.049
31	AMW4891*272	71	83	158.167	1-Octanol, 3,7-dimethyl-	106-21-8	1194	84.4	0.250	0.523	0.411	0.023
32	AMW0660*272	71	83	158.167	2,7-dimethyl-1-Octanol	15250-22-3	1187	82.93	0.238	0.506	0.390	0.022
33	AMW0774	127	85	184.219	Nonane, 5-methyl-5-propyl-	17312-75-3	1202	81.1	0.029	0.055	0.043	0.022
34	AMW4652	121	107	150.141	Artemisia triene	18383-70-5	923	32.65	0.001	0.002	0.002	0.032
35	AMW4560	67	83	112.125	Cyclopropane,	6976-27-8	798	70.44	0.120	0.056	0.137	0.244

					(1,2-dimethylpropyl)-							
36	AMW3514	85	70	141.115	Cyclobutylamine, N-(2-methylpropionyl)-	1339757-02-6	1206	72.95	0.198	0.381	0.301	0.024
37	AMW2140 *322	91	92	136.125	.alpha.-Pinene	80-56-8	936. 3588 9410 5373	81.39	0.003	0.005	0.005	0.031
38	AMW2441 *322	91	92	136.125	(1R)-2,6,6-Trimethylbicyclo[3.1.1] hept-2-ene	7785-70-8	936. 4724 2455 7752 4	81.39	0.003	0.005	0.005	0.031
39	AMW2362 *322	91	92	136.125	(1S)-2,6,6-Trimethylbicyclo[3.1.1] hept-2-ene	7785-26-4	936. 5244 5369 4068 6	81.39	0.003	0.005	0.005	0.031
40	AMW3459	114	44	114.079	Acetamide, N-[(dimethylamino)methylidene]-	1000197-09-8	1194	76.35	0.004	0.007	0.005	0.022
41	AMW2283	57	55	128.12	2,2-dimethyl-Hexanal	996-12-3	993	68.76	0.027	0.047	0.040	0.033
42	AMW2803	71	41	214.157	Pentanoic acid, 2-methyl-, anhydride	63169-61-9	1348	75.21	0.013	0.030	0.019	0.065
43	AMW0778 *369	84	71	184.219	Undecane, 5,7-dimethyl-	17312-83-3	1190	80.12	0.051	0.095	0.079	0.022
44	AMW0880	41	39	202.121	Ethanedioic acid, dibutyl ester	2050-60-4	1323	68.75	0.006	0.014	0.009	0.055
45	AMW3337	83	97	158.167	1-Hexanol, 5-methyl-2-(1-methylethyl)-	2051-33-4	1146	75.27	0.003	0.002	0.005	0.160
46	AMW4317	72	55	172.074	Oxalic acid, cyclobutyl ethyl ester	1000309-69-3	1202	66.29	0.015	0.030	0.023	0.026
47	AMW0911	98	93	154.136	cis-7-Decen-1-al	21661-97-2	1199	80.58	0.048	0.086	0.069	0.033
48	AMW2413	141	140	198.235	Tridecane, 5-methyl-	25117-31-1	1348	85.88	0.010	0.021	0.013	0.062
49	AMW1700 *132	99	55	198.235	Dodecane, 4,6-dimethyl-	61141-72-8	1325	85.62	0.015	0.031	0.020	0.068
50	AMW2648	105	131	132.058	1H-Inden-1-one, 2,3-dihydro-	83-33-0	1292	66.46	0.001	0.002	0.002	0.044
51	AMW2852 *308	70	42	184.219	Decane, 6-ethyl-2-methyl-	62108-21-8	1204	82.29	0.041	0.111	0.087	0.009
52	AMW2535 *308	70	42	184.219	Undecane, 4,8-dimethyl-	17301-33-6	1214	82.29	0.041	0.111	0.087	0.009
53	AMW4953	112	85	130.027	Butanedioic acid, methylene-	97-65-4	1306	74.13	0.003	0.005	0.004	0.079
54	AMW1944	80	79	136.125	2-Methyl-1-nonene-3-yne	70058-00-3	1002	73.61	0.002	0.003	0.003	0.043
55	AMW4356	93	91	122.11	3-Ethylidenecycloheptene	1000211-16-7	1012	75.78	0.009	0.013	0.012	0.022
56	AMW2801	54	55	143.095	2-(E)-Hexenoic acid, (4S)-amino-5-methyl-	267008-79-7	1317	69.58	0.002	0.001	0.002	0.125
57	AMW1274	81	53	110.073	Furan, 2-propyl-	4229-91-8	791	64.62	0.023	0.011	0.025	0.257
58	AMW0490	85	42	86.048	2-Imidazolidinone	120-93-4	1331	74.19	0.027	0.057	0.036	0.061
59	AMW3763	69	99	99.032	Pyrrolidine-2,4-dione	37772-89-7	1195	75.42	0.506	0.252	0.352	0.240
60	AMW4708	107	121	122.084	2-Isopropylpyrazine	29460-90-0	988	78.51	0.006	0.007	0.007	0.033
61	AMW1292	70	68	139.1	9-Azabicyclo[3.3.1]nonan-3-one	4390-39-0	1317	75.95	0.007	0.017	0.011	0.069
62	AMW2528	169	93	168.079	1,2,3-Trimethoxybenzene	634-36-6	1313	66.06	0.007	0.013	0.006	0.035
63	AMW1250	97	98	142.063	3(2H)-Furanone, 4-methoxy-2,5-dimethyl-	4077-47-8	1057	82.67	0.008	0.005	0.010	0.166
64	AMW2120	111	153	168.151	(2,2,6-Trimethyl-bicyclo[4.1.0]he pt-1-yl)-methanol	78996-11-9	1325	80.49	0.014	0.029	0.019	0.061
65	AMW2240 *058	105	79	106.078	o-Xylene	95-47-6	894. 2110 1774 0429	64.04	0.012	0.011	0.010	0.004
66	AMW0671	86	43	171.162	N,N-dibutyl-Acetamide	1563-90-2	1336	72	0.002	0.004	0.002	0.059
67	AMW2902	69	41	97.028	1H-1,2,3-Triazole-4-carboxaldehy de	16681-68-8	1314	71.72	0.021	0.047	0.031	0.064
68	AMW4160	127	126	198.235	Dodecane, 2,5-dimethyl-	56292-65-0	1312	79.01	0.001	0.002	0.001	0.081
69	AMW1291	71	57	226.266	Nonane, 2,2,4,4,6,8,8-heptamethyl-	4390-04-9	1322	73.97	0.080	0.163	0.106	0.066
70	AMW1282	69	83	154.172	pentyl-Cyclohexane	4292-92-6	1136	80.64	0.002	0.001	0.002	0.027
71	AMW4053	71	55	200.105	Oxalic acid, allyl pentyl ester	1000309-23-2	1331	86.21	0.039	0.085	0.054	0.061
72	AMW4999	55	70	182.167	1-Dodecyn-4-ol	74646-36-9	1400	75.95	0.018	0.042	0.026	0.051
73	AMW4049	68	41	128.095	(3R)-(+)-3-Acetamidopyrrolidine	1000136-85-4	1318	71.34	0.001	0.003	0.002	0.044

74	AMW0719	79	109	138.104	1-(1-cyclohexen-1-yl)-1-Propanone	1655-03-4	1162	71.82	0.005	0.003	0.009	0.181
75	AMW3041	69	112	112.027	5-Methylenehydantoin	7673-65-6	1331	71.2	0.004	0.009	0.006	0.054
76	AMW2556	55	42	202.121	Oxalic acid, pentyl propyl ester	1000309-25-7	1340	71.25	0.002	0.005	0.004	0.062
77	AMW4442	98	41	143.095	Cyclohexanecarboxylic acid, 1-amino-	2756-85-6	1394	76.4	0.011	0.025	0.015	0.054
78	AMW4457	41	39	170.058	Cyclopropanebutanoic acid, 2,4-dioxo-, methyl ester	167408-67-5	1390	69.66	0.014	0.032	0.020	0.052
79	AMW1822	84	57	198.235	Tetradecane	629-59-4	1400	91.31	0.012	0.029	0.018	0.046
80	AMW1065	77	93	136.125	2-methyl-5-propan-2-ylbicyclo[3.1.0]hex-2-ene	2867-05-2	929	78.14	0.002	0.003	0.003	0.004
81	AMW3131	57	43	144.053	Propanoic acid, 2-acetylhydrazono-	64096-81-7	1384	65.29	0.116	0.272	0.165	0.052
82	AMW3007	57	56	200.105	Oxalic acid, butyl cyclobutyl ester	1000309-69-4	1390	75.74	0.117	0.274	0.166	0.053
83	AMW0283 *132	99	55	198.235	3,5-Dimethyldodecane	107770-99-0	1321	80.34	0.003	0.007	0.004	0.061
84	AMW1006 *158	82	65	152.12	(2E,4Z)-2,4-Decadienal	25152-83-4	1295	79.23	0.002	0.002	0.004	0.145
85	AMW0956 *158	82	65	152.12	2,4-Decadienal	2363-88-4	1319<br>.205<br>2980<br>1325	79.23	0.002	0.002	0.004	0.145
86	AMW1007 *158	82	65	152.12	2,4-Decadienal, (E,E)-	25152-84-5	1320<br>.077	79.23	0.002	0.002	0.004	0.145
87	AMW0399	71	67	196.219	1-Tetradecene	1120-36-1	1392	81.89	0.089	0.207	0.126	0.052
88	AMW0877	58	71	128.12	1-butyl-Cyclobutanol	20434-34-8	1011	82.31	0.001	0.002	0.002	0.030
89	AMW1228 *135	57	56	212.25	Dodecane, 2,6,10-trimethyl-	3891-98-3	1366	79.93	0.006	0.011	0.007	0.064
90	AMW2014 *135	57	56	212.25	2,7,10-trimethyl-Dodecane	74645-98-0	1371	79.93	0.006	0.011	0.007	0.064
91	AMW0487	53	82	182.131	(E)-Hex-3-enyl (E)-2-methylbut-2-enoate	120603-00-1	1317	69.84	0.001	0.002	0.003	0.313
92	AMW4993	55	43	200.178	Hexanoic acid, hexyl ester	6378-65-0	1384	68.01	0.001	0.002	0.002	0.093
93	AMW1140	70	41	156.115	.delta.-Nonalactone	3301-94-8	1396	71.86	0.017	0.040	0.025	0.051
94	AMW0571 *326	91	105	136.125	3-Carene	13466-78-9	1012<br>.459<br>7207<br>304	74.52	0.003	0.005	0.004	0.019
95	AMW3273 *326	91	105	136.125	2-Carene	554-61-0	1001	74.52	0.003	0.005	0.004	0.019
96	AMW1080 *326	91	105	136.125	(+)-4-Carene	29050-33-7	1009	74.52	0.003	0.005	0.004	0.019
97	AMW4996	72	56	128.095	4-Piperidinecarboxamide	39546-32-2	1398	78.24	0.005	0.012	0.007	0.056
98	AMW2378 *293	99	85	212.25	Tridecane, 4,8-dimethyl-	55030-62-1	1401	86.32	0.017	0.040	0.024	0.049
99	AMW2523 *293	99	85	212.25	Tridecane, 2,5-dimethyl-	56292-66-1	1409	86.32	0.017	0.040	0.024	0.049
100	AMW3715	100	44	128.022	1-Methyl-2,4,5-trioxoimidazolidine	3659-97-0	1324	73.23	0.001	0.002	0.002	0.072
101	AMW0582	43	41	198.162	6,7-Dodecanedione	13757-90-9	1392	75.25	0.052	0.121	0.075	0.053
102	AMW4961	83	95	166.099	Bicyclo[2.2.1]heptane-2,3-dione, 1,7,7-trimethyl-, (1S)-	2767-84-2	1319	70.32	0.013	0.027	0.017	0.066
103	AMW2938	112	113	198.235	Tridecane, 7-methyl-	26730-14-3	1345	77.93	0.001	0.002	0.001	0.074
104	AMW3431	93	147	202.172	(3R,4aS,8aS)-8a-Methyl-5-methylene-3-(prop-1-en-2-yl)-1,2,3,4,4a,5,6,8a-octahydronaphthalene	212394-95-1	1477	75.5	0.009	0.000	0.000	0.030
105	AMW1420	109	69	154.136	4-methyl-1-(1-methylethyl)-Bicyclo[3.1.0]hexan-3-ol	513-23-5	1168	75.72	0.001	0.000	0.003	0.155
106	AMW4337	42	41	112.075	4-Amino-3,5-dimethyl-1,2,4-triazole	3530-15-2	1398	74.42	0.005	0.013	0.008	0.053
107	AMW2715	67	58	114.104	Cyclohexanol, 4-methyl-, trans-	7731-29-5	964	81.82	0.005	0.002	0.003	0.003
108	AMW3008	44	43	104.011	Propanedioic acid	141-82-2	1198	83.4	0.008	0.016	0.012	0.023
109	AMW0756 *119	57	70	184.219	Undecane, 2,9-dimethyl-	17301-26-7	1233	81.1	0.004	0.008	0.006	0.043
110	AMW3025	55	56	84.044	1H-Tetrazole, 5-methyl-	4076-36-2	1184	80.02	0.003	0.007	0.005	0.028
111	AMW0712	81	110	138.141	1,9-Decadiene	1647-16-1	964	70.53	0.034	0.015	0.032	0.499
112	AMW0290	69	68	86.037	(E)-2-Butenoic acid	107-93-7	894	67.25	0.010	0.006	0.014	0.150

113	AMW3033	77	81	138.104	2-Cyclohexen-1-one, 4,4,5-trimethyl-	17429-29-7	1157	72.65	0.001	0.001	0.002	0.242
114	AMW1251	53	80	164.131	3-butyl-2,5-dimethyl-Pyrazine	40790-29-2	1263	70.31	0.001	0.001	0.001	0.147
115	AMW0533	84	55	128.12	Octanal	124-13-0	1003 .276 0472 6101	90.61	0.042	0.026	0.050	0.144
116	AMW3148	68	127	169.11	2-Cyano-2-isopropyl-3-methylbu tanoic acid	866-29-5	1326	75.78	0.002	0.003	0.002	0.054
117	AMW4684 *211	55	56	100.052	2(3H)-Furanone, dihydro-4-methyl-	1679-49-8	960	78.73	0.022	0.013	0.026	0.082
118	AMW4686 *211	55	56	100.052	2H-Pyran-2-one, tetrahydro-	542-28-9	961	78.73	0.022	0.013	0.026	0.082
119	AMW2516	45	41	116.12	2-Heptanol	543-49-7	900. 3611 9711 0423 2	72.73	0.024	0.015	0.033	0.201
120	AMW2646	67	71	226.118	1-Octyl trifluoroacetate	2561-21-9	1059	87.15	0.008	0.005	0.010	0.245
121	AMW2131	41	40	97.039	1,2,4,5-Tetrazin-3-Amine	79329-74-1	1151	86.08	0.011	0.005	0.021	0.169
122	AMW0828 *226	57	55	112.089	(E)-2-Heptenal	18829-55-5	958	79.97	0.009	0.005	0.011	0.145
123	AMW4681 *226	57	55	112.089	2-Heptenal, (Z)-	57266-86-1	958	79.97	0.009	0.005	0.011	0.145
124	AMW0166	121	107	228.051	2-(4-bromobutyl)-5,5-dimethyl-1, 3-Cyclopentadiene	1010163-68-7	1359	68.8	0.001	0.001	0.002	0.167
125	AMW4696	83	69	140.157	Nonane, 3-methylene-	51655-64-2	973	81.5	0.009	0.006	0.009	0.041
126	AMW2143	69	57	116.12	1-Hexanol, 4-methyl-	818-49-5	953	83.47	0.008	0.005	0.010	0.198
127	AMW0561	69	57	116.12	3-methyl-1-Hexanol	13231-81-7	895	75.79	0.011	0.007	0.016	0.134
128	AMW4668	68	41	138.141	3-Ethyl-1,5-octadiene	1000114-87-7	947	68.8	0.007	0.004	0.009	0.133
129	AMW0829 *256	55	96	140.12	2-Nonenal, (E)-	18829-56-6	1161 .205 9765 2081	95.06	0.026	0.013	0.051	0.146
130	AMW1691 *256	55	96	140.12	2-Nonenal, (Z)-	60784-31-8	1148	95.06	0.026	0.013	0.051	0.146
131	AMW0986 *256	55	96	140.12	2-Nonenal	2463-53-8	1161	95.06	0.026	0.013	0.051	0.146
132	AMW4680	70	69	168.188	5-Undecene, 9-methyl-, (Z)-	74630-65-2	957	74.79	0.007	0.004	0.009	0.175
133	AMW1535 *254	67	68	138.104	2,6-Nonadienal, (E,Z)-	557-48-2	1154 .749 1995 7311	74.6	0.009	0.005	0.018	0.114
134	AMW2457 *254	67	68	138.104	2,6-Nonadienal, (E,E)-	17587-33-6	1153	74.6	0.009	0.005	0.018	0.114
135	AMW0217	56	41	128.084	2(3H)-Furanone, dihydro-5-propyl-	105-21-5	1157	76.51	0.009	0.004	0.017	0.159
136	AMW4827	95	41	152.157	Naphthalene, decahydro-2-methyl-	2958-76-1	1129	78.84	0.002	0.001	0.003	0.165
137	AMW5149	81	69	264.209	2,6,10-Dodecatrien-1-ol, 3,7,11-trimethyl-, acetate, (E,E)-	4128-17-0	1843	59.62	0.004	0.000	0.000	2.3E-05
138	AMW0623	111	41	154.147	Azacyclohexane, 3-[1-pyrrolidyl]-	144243-28-7	1339	72.7	0.001	0.003	0.002	0.065
139	AMW0936	55	82	110.048	4(1H)-Pyrimidinone, 1-methyl-	2228-30-0	1344	74.7	0.005	0.009	0.007	0.102
140	AMW2084	71	83	212.214	Vinyl lauryl ether	765-14-0	1494	77.56	0.015	0.032	0.017	0.102
141	AMW4861	70	83	168.188	2-Undecene, 6-methyl-, (Z)-	74630-43-6	1162	84.81	0.029	0.014	0.059	0.157
142	AMW3525	109	110	152.157	5-Cyclohexyl-1-pentene	5729-54-4	1124	80.88	0.002	0.001	0.002	0.087
143	AMW0910	95	94	110.073	1-Methoxy-1,4-cyclohexadiene	2886-59-1	960	76.36	0.001	0.001	0.002	0.200
144	AMW0133	111	43	126.091	1-Ethyl-1H-pyrazole-3,4-diamine	1000460-38-8	1364	79.07	0.001	0.001	0.001	0.090
145	AMW0394	72	43	134.094	Ethanol, 2-(2-ethoxyethoxy)-	111-90-0	1007	62.85	0.008	0.006	0.011	0.121
146	AMW3469	70	69	156.115	Amyl crotonate	1000429-17-2	1147	69.85	0.002	0.001	0.003	0.209
147	AMW4068	84	112	113.059	Creatinine	60-27-5	1494	67.2	0.003	0.005	0.003	0.123
148	AMW4637	67	69	112.089	4-Heptyn-2-ol	19781-81-8	909	73.73	0.011	0.005	0.020	0.146
149	AMW1105	67	109	124.089	3,4,4-trimethyl-2-Cyclopenten-1-one	30434-65-2	1129	77.65	0.003	0.001	0.005	0.060
150	AMW1412	85	43	142.074	Sarcosine anhydride	5076-82-4	1468	69.63	0.004	0.009	0.005	0.099
151	AMW0658	81	96	152.157	1-pentyl-Cyclohexene	15232-85-6	1159	72.89	0.010	0.004	0.019	0.158
152	AMW2381	98	126	198.235	Tridecane, 6-methyl-	13287-21-3	1344	80.13	0.001	0.002	0.002	0.102

					Ethanol,				0.034	0.023	0.043	0.131
153	AMW3998	44	43	132.079	2-[2-(ethenyloxy)ethoxy]-	929-37-3	997	69.46				
154	AMW2009	57	42	168.188	1-Undecene, 9-methyl-	74630-41-4	1152	85.82	0.012	0.004	0.024	0.162
155	AMW2836	57	43	226.247	Borane, diethyl(decyloxy)-	1000152-34-3	1522	71.11	0.002	0.005	0.002	0.120
156	AMW4307	69	41	110.048	4(1H)-Pyrimidinone, 2-methyl-	19875-04-8	1370	82.85	0.003	0.002	0.005	0.114
	AMW0987								0.002	0.002	0.004	0.096
157	*310	84	70	168.151	2-Undecenal	2463-77-6	1367	86.08				
	AMW1456								0.002	0.002	0.004	0.096
158	*310	84	70	168.151	2-Undecenal, E-	53448-07-0	1365	86.08				
	AMW4849				Cyclohexanol,				0.004	0.002	0.006	0.154
159	*270	94	79	154.136	1-methyl-4-(1-methylethenyl)-	138-87-4	1153	78.8				
160	AMW3756	85	57	128.059	2,5-Piperazinedione, 3-methyl-	6062-46-0	1592	71.24	0.006	0.013	0.007	0.125
					2-methoxy-6-methyl-4H-Pyran-4-				0.001	0.001	0.002	0.221
161	AMW1273	53	43	140.047	one	4225-42-7	1295	69.13				
					Oxalic acid, cyclobutyl hexyl				0.005	0.009	0.005	0.141
162	AMW3574	43	41	228.136	ester	1000309-69-7	1589	66.82				
					2-Ethylhexanal ethylene glycol				0.001	0.001	0.001	0.394
163	AMW3051	73	45	172.146	acetal	1000431-01-2	1196	76.59				
164	AMW1729	86	42	101.084	N,N-diethyl-Formamide	617-84-5	921	70.74	0.004	0.005	0.009	0.060
165	AMW1022	79	57	180.188	1-Tridecyne	26186-02-7	1297	82.38	0.002	0.001	0.002	0.274
					Sulfurous acid, 2-ethylhexyl				0.010	0.022	0.011	0.119
166	AMW0055	57	55	250.16	isobutyl ester	1000309-18-7	1601	73.87				
					1,3-Diazabicyclo[3.2.2]nonan-4-o				0.003	0.002	0.005	0.097
167	AMW3628	82	41	140.095	ne	89851-63-8	1376	73.17				
					Isobutyric acid,				0.014	0.005	0.025	0.168
168	AMW4472	43	41	217.967	2,2,2-trichloroethyl ester	1000354-63-4	1153	68.84				
169	AMW1570	122	81	140.12	3,6-Nonadien-1-ol, (E,Z)-	56805-23-3	1153	76.46	0.002	0.001	0.004	0.155
170	AMW0181	70	41	141.152	4-Propylcyclohexylamine	102653-37-2	1138	71.89	0.001	0.001	0.002	0.150
					Butanoic acid, 3-methyl-,				0.006	0.003	0.010	0.182
171	AMW3456	85	60	144.115	1-methylethyl ester	32665-23-9	904	71.19				
	AMW1099								0.026	0.009	0.042	0.256
172	*069	95	55	124.089	3,5-Octadien-2-one, (E,E)-	30086-02-3	1073	68.12				
173	AMW4864	93	39	136.052	Ethanone, 1-(2-hydroxyphenyl)-	118-93-4	1163	56.29	0.006	0.003	0.012	0.150
174	AMW3587	43	41	70.028	1H-Tetrazole	288-94-8	1133	82.95	0.002	0.002	0.005	0.100
175	AMW1395	66	67	94.053	4-Aminopyridine	504-24-5	1158	61.37	0.001	0.000	0.002	0.179
	AMW2159								0.018	0.007	0.033	0.153
176	*122	69	57	168.188	6-methyl-5-Undecene	83687-45-0	1162	82.65				
	AMW1703								0.018	0.007	0.033	0.153
177	*122	69	57	168.188	4-methyl-4-Undecene	61142-40-3	1165	82.65				
	AMW2012								0.018	0.007	0.033	0.153
178	*122	69	57	168.188	(Z)-4-methyl-4-Undecene	74630-57-2	1165	82.65				
179	AMW0841	43	41	114.104	3-methyl-Hexanal	19269-28-4	910	85.64	0.053	0.027	0.088	0.158
					4H-1,2,4-Triazol-3-amine,				0.008	0.020	0.010	0.119
180	AMW3788	43	126	126.091	4-propyl-	58661-97-5	1540	84.51				
181	AMW1141	71	41	188.141	3-Hydroxydecanoic acid	33044-91-6	1545	72.44	0.006	0.018	0.008	0.113
182	AMW4596	41	39	102.068	1-Hydroxy-2-pentanone	64502-89-2	858	75.83	0.020	0.011	0.022	0.161
	AMW1181								0.094	0.039	0.183	0.158
183	*057	70	67	138.141	3,3,6-trimethyl-1,5-Heptadiene	35387-63-4	904	75.26				
	AMW2005								0.094	0.039	0.183	0.158
184	*057	70	67	138.141	3,3,5-trimethyl-1,5-Heptadiene	74630-29-8	919	75.26				
185	AMW0918	55	97	116.084	6-hydroxy-2-Hexanone	21856-89-3	1037	71.06	0.026	0.019	0.040	0.272
					1-(1-methylethyl)-2-nonyl-Cyclo				0.002	0.004	0.002	0.067
186	AMW1269	69	56	210.235	propane	41977-39-3	1468	73.58				
							911.		0.002	0.001	0.004	0.168
							1632					
							7595					
187	AMW0469	95	43	110.037	Ethanone, 1-(2-furanyl)-	1192-62-7	2008	57.49				
188	AMW2851	57	85	85.053	N-Vinylacetamide	5202-78-8	885	77.27	0.002	0.002	0.004	0.031
189	AMW2854	57	69	240.282	Tetradecane, 2,6,10-trimethyl-	14905-56-7	1539	85.79	0.016	0.041	0.020	0.112
	AMW1865								0.092	0.039	0.178	0.185
190	*062	70	56	126.141	(E)-2-Nonene	6434-78-2	897	72.77				
	AMW0859								0.092	0.039	0.178	0.185
191	*062	70	56	126.141	3-Nonene	20063-77-8	888	72.77				
	AMW0865								0.092	0.039	0.178	0.185
192	*062	70	56	126.141	cis-3-Nonene	20237-46-1	888	72.77				
	AMW0919								0.092	0.039	0.178	0.185
193	*062	70	56	126.141	4-Nonene	2198-23-4	888	72.77				
194	AMW0532	70	56	126.141	1-Nonene	124-11-8	889	72.77	0.092	0.039	0.178	0.185

	*062											
	AMW0933								0.092	0.039	0.178	0.185
195	*062	70	56	126.141	2-Nonene	2216-38-8	897	72.77				
196	AMW3274	41	67	128.084	CH3C(O)O(CH2)3CH=CH2	1576-85-8	896	73.84	0.023	0.009	0.044	0.153
							893.		0.042	0.018	0.085	0.155
							5575					
							5566					
197	AMW0320	55	70	98.073	Cyclohexanone	108-94-1	0824	59.43				
					1H-Imidazole,				0.008	0.005	0.012	0.244
198	AMW1454	54	42	84.069	4,5-dihydro-2-methyl-	534-26-9	900	71.11				
199	AMW2920	112	140	226.266	Pentadecane, 7-methyl-	6165-40-8	1541	81.4	0.002	0.004	0.002	0.125
					4-Octen-3-one,				0.001	0.002	0.001	0.041
200	AMW3250	97	41	170.131	6-ethyl-7-hydroxy-	78464-96-7	1334	74.88				
201	AMW4981	97	69	160.11	2-Hydroxyoctanoic acid	617-73-2	1367	74.76	0.002	0.002	0.005	0.106

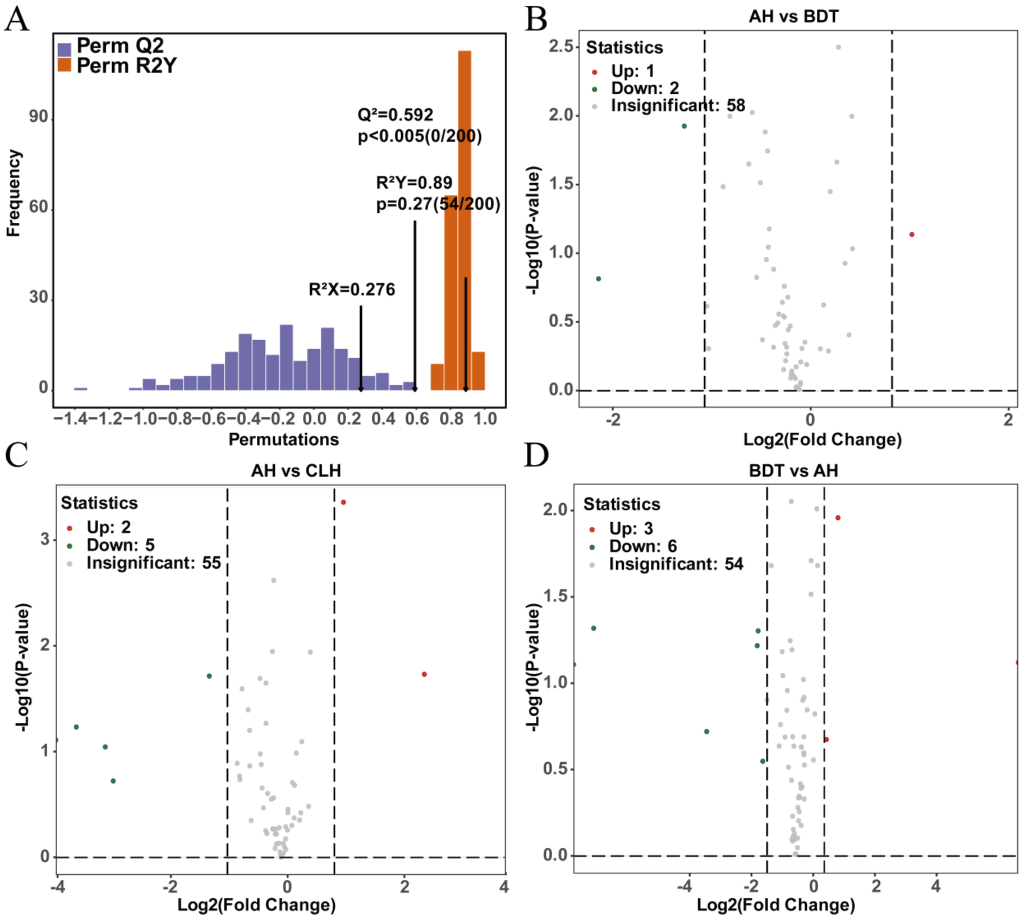

Figure S1. Multivariate model validation and differential analysis of free amino acids among sheep breeds. (A) Permutation test of the OPLS-DA model for free amino acid profiles among the three sheep breeds. (B–D) Volcano plots showing differential free amino acids in the pairwise comparisons of AH vs BDT (B), AH vs CLH (C), and BDT vs CLH (D).

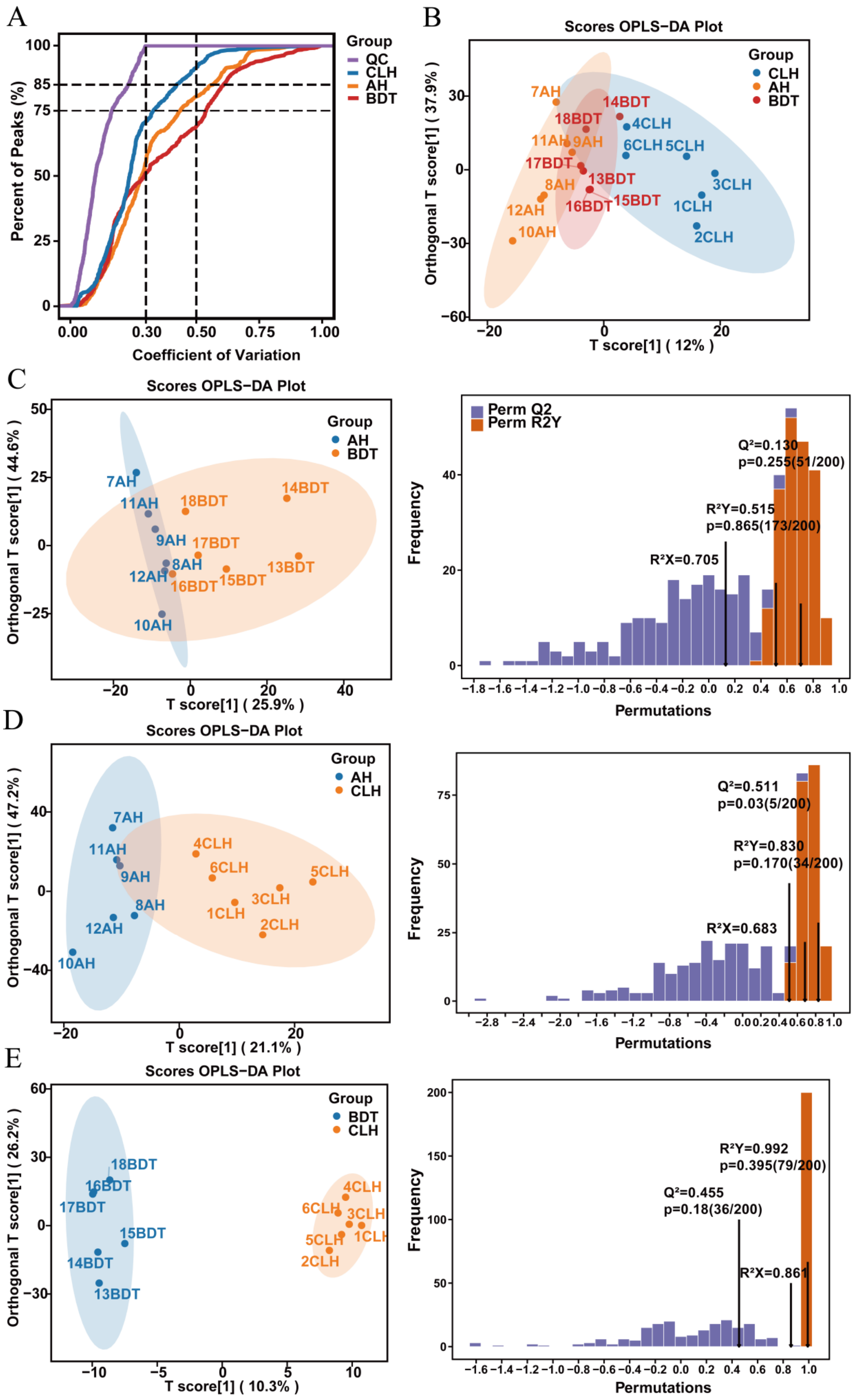

Figure S2 Quality assessment and OPLS-DA of volatile organic compound profiles among

sheep breeds. (A) Distribution of coefficients of variation (CVs) for volatile organic compounds (VOCs) in quality control (QC) samples. (B) OPLS-DA score plot of VOC profiles among the three sheep breeds. (C–E) OPLS-DA scoring plot and permutation test for pairwise comparison of AH and BDT (C), AH and CLH (D), and BDT and CLH (E).
